# Associations Between Neuromuscular Fatigue, Anxiety, and Peripheral Biomarkers of Neuronal Injury, Inflammation, and Mitochondrial Dysfunction in Treatment‐Naïve Relapsing‐Remitting Multiple Sclerosis

**DOI:** 10.1002/brb3.71707

**Published:** 2026-08-25

**Authors:** Amanda V. Steckert, Keli Dias Feltrin, Luiz Fernando Silva Rodrigues, Felipe Moreira Liz, Maria Milena Figueiredo Muller, Daniel Paulo Bortoluzzi, Andréia Biolchi Mayer, Luciane Bisognin Ceretta, Tatiana Barichello, Felipe Dal‐Pizzol, Diogo Dominguini

**Affiliations:** ^1^ Graduate Program in Health Management University of Extreme South of Santa Catarina Criciúma Brazil; ^2^ Graduation in Nursing University of Extreme South of Santa Catarina (UNESC) Criciúma Brazil; ^3^ Laboratory of Experimental Pathophysiology, Graduate Program in Health Sciences University of Extreme South of Santa Catarina Criciúma Brazil; ^4^ Graduate Program in Environment and Health University of Planalto Catarinense (UNIPLAC) Lages Brazil; ^5^ Faillace Department of Psychiatry and Behavioral Sciences, McGovern Medical School The University of Texas Health Science Center At Houston (UTHealth) Houston USA

**Keywords:** cognitive alterations, fatigue, neurological diseases

## Abstract

**Objective:**

To investigate whether neuromuscular fatigue and anxiety are associated with a peripheral biological signature involving inflammation, oxidative stress, mitochondrial homeostasis, neurotrophic support, and neuronal/glial injury markers in treatment‐naïve patients with relapsing‐remitting multiple sclerosis (RRMS).

**Method:**

This cross‐sectional case‐control study included 20 newly diagnosed, treatment‐naïve patients with RRMS and 20 age‐ and sex‐matched healthy controls. Anxiety symptoms were assessed using the Beck Anxiety Inventory, while fatigue was evaluated using the Modified Fatigue Impact Scale and the Fatigue Severity Scale. Peripheral blood samples were collected before the initiation of multiple sclerosis‐specific therapy. Cytokines, neurotrophic factors, neuronal/glial injury markers, vitamin D, oxidative damage parameters, mitochondrial reactive oxygen species, mitochondrial DNA damage, inflammasome‐related proteins, apoptotic markers, and mitochondrial homeostasis‐related genes were analyzed in serum using ELISA, biochemical assays, Western blotting, and RT‐PCR.

**Results:**

Compared with healthy controls, patients with RRMS showed higher anxiety and fatigue scores, increased circulating IL‐1β, IL‐18, and TNF‐α levels, and increased NLRP3 inflammasome expression. These alterations were accompanied by increased oxidative damage, higher mitochondrial reactive oxygen species production, increased mitochondrial 8‐oxoG levels, increased Bax expression, reduced Bcl‐2 expression, decreased TFAM expression, and increased Pink‐1 and Parkin expression. In addition, patients exhibited increased serum levels of S100β and neuron‐specific enolase, along with reduced brain‐derived neurotrophic factor (BDNF) and nerve growth factor (NGF) levels. Exploratory correlation analyses suggested biologically plausible associations among neurotrophic signaling, mitochondrial biogenesis, inflammatory activation, oxidative stress, and markers of neuronal/glial injury.

**Conclusion:**

These findings suggest that treatment‐naïve RRMS is characterized by a peripheral inflammatory–mitochondrial imbalance associated with fatigue, anxiety, increased neuronal/glial injury markers, and reduced neurotrophic support. Peripheral biomarkers such as S100β, neuron‐specific enolase, BDNF, and mitochondrial homeostasis‐related markers may represent accessible candidate indicators of disease‐related biological alterations.

## Graphical abstract

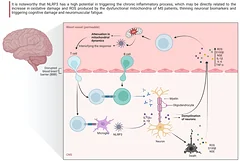

## 1 Introduction

The nervous system plays a central role in maintaining essential human functions, including perception, movement, learning, memory, and emotional regulation. Its proper functioning depends on the coordinated activity of billions of neurons that communicate through complex chemical and electrical synapses, ensuring physiological and cognitive homeostasis (Stadelmann et al. 2019). Disruptions in these neural processes can compromise central nervous system integrity and contribute to the development of neurological disorders such as multiple sclerosis (MS), a chronic autoimmune and neuroinflammatory disease characterized by demyelination, axonal damage, and progressive neurological dysfunction (Reich et al., 2018; Stadelmann et al., 2019).

Multiple sclerosis predominantly affects young adults, especially between 20 and 40 years of age, and is associated with substantial physical disability, reduced quality of life, and decreased life expectancy (Walton et al., 2020; Oliva Ramirez et al., 2021). Current evidence indicates that the development and progression of MS result from a complex interaction between genetic susceptibility and environmental factors, including vitamin D deficiency, smoking, viral infections, obesity, and lifestyle habits, all of which may influence immune dysregulation and disease progression (Murdaca et al. 2019; Oliva Ramirez et al. 2021). Disease onset is often unpredictable, and progressive neurological damage may lead to significant long‐term disability and severe clinical complications.

The global prevalence of MS has increased substantially in recent decades, currently affecting approximately 2.8 million people worldwide (Yamout et al., 2018; Yi et al., 2024). Women are more frequently affected than men, with a female‐to‐male ratio of nearly 2:1, suggesting important hormonal and immunological influences in disease susceptibility (D'Anca et al., 2023). Higher prevalence rates are reported in North America, Europe, Australia, and New Zealand, while Latin American countries, including Brazil, show lower but progressively increasing prevalence rates. In Brazil, MS represents an important public health concern, particularly among economically active adults, with increasing hospitalization rates observed after adolescence and a higher concentration of cases between 20 and 49 years of age (Howard et al., 2020; Yi et al. 2024). These epidemiological trends reinforce the need for better understanding of the biological and clinical factors involved in disease progression and symptom burden.

The clinical manifestations of MS vary according to the location and extent of central nervous system lesions, resulting in a broad spectrum of neurological symptoms. Common manifestations include limb weakness, impaired coordination, ataxia, paresthesia, tremors, visual disturbances such as diplopia, and difficulties in writing and motor control (Stadelmann et al. 2019). In addition to motor and sensory symptoms, many patients experience autonomic dysfunction, including bladder, bowel, and sexual disturbances, as well as speech impairment and significant cognitive decline, which can substantially affect daily functioning and quality of life (Morrow, 2024; Gómez‐Melero et al., 2024). Among the non‐motor manifestations, anxiety and fatigue are particularly prevalent and debilitating. Anxiety disorders affect approximately 20%–40% of people with MS, substantially higher than the general population, and are associated with reduced quality of life and treatment adherence (Rossi et al. 2017; Boeschoten et al. 2017). Fatigue, reported by 75%–90% of MS patients, is often cited as the most disabling symptom, interfering with employment, social functioning, and daily activities even in the absence of significant physical disability (Reich et al., 2018). Unlike fatigue in healthy individuals, MS‐related fatigue is disproportionate to activity level, worsens with heat, and does not reliably improve with rest. The severity of these neuropsychiatric symptoms often correlates poorly with physical disability measures, suggesting distinct underlying pathophysiological mechanisms that warrant specific investigation (Heitmann et al., 2020; Yamout et al., 2018; Dobryakova et al., 2015).

The disease is marked by axonal damage and demyelination triggered by an immune response within the CNS. This response involves microglial activation and the participation of CD4+ and CD8+ T cells, macrophages, natural killer cells, and several pro‐inflammatory mediators, ultimately disrupting efficient communication between the brain and peripheral organs (Olcum et al. 2020). Active MS lesions display a predominantly Th1/Th17‐driven immune profile, promoting the release of cytokines such as IL‐1, IL‐2, IL‐17, IL‐22, TNF‐α, and IFN‐γ, which contribute to blood–brain barrier disruption, demyelination, and neuronal injury (Dargahi et al. 2017; Bigaut et al., 2019). These inflammatory mechanisms are closely linked not only to disease progression, but also to fatigue severity and cognitive dysfunction. Beyond structural demyelination and axonal injury, the inflammatory milieu in MS generates a cascade of soluble mediators that profoundly affect neural function. Proinflammatory cytokines—including IL‐1β, IL‐6, TNF‐α, and IFN‐γ—are elevated in the cerebrospinal fluid and CNS parenchyma of MS patients and have been directly implicated in the pathogenesis of neuropsychiatric symptoms (Rossi et al. 2017; Chalah and Ayache, 2017; Howard et al., 2020). These cytokines can cross the blood–brain barrier, modulate neurotransmitter systems, and activate resident CNS immune cells, thereby linking peripheral immune activation to central nervous system dysfunction (Dantzer et al. 2008; Miller et al. 2013). Notably, intrathecal inflammation—even when subclinical on conventional MRI—has been associated with elevated anxiety and depressive symptom scores in treatment‐naïve relapsing‐remitting MS patients, with scores declining as neuroinflammation subsided (Rossi et al. 2017). This observation supports a direct mechanistic link between CNS inflammatory activity and affective symptomatology, independent of overt clinical relapse or accumulated disability.

Several genes have been implicated in MS susceptibility, including MHC class II variants—particularly the HLA‐DRB1*1501 allele—and, to a lesser degree, genes related to IL‐7 and IL‐2 receptors, cytokines and chemokines (CXCR5, IL‐12A, IL‐12β, and IL‐12Rβ1), as well as genes coding for costimulatory molecules such as CD80, CD86, and CD37 (Wessels and Rink 2020; Restuccia et al. 2025). The precise contribution of these genes to immune regulation and their interaction with environmental triggers, however, is still not fully understood. Among these environmental factors, sunlight exposure stands out due to its role in vitamin D synthesis. Evidence suggests that reduced vitamin D production may increase MS risk, as reported by Murdaca et al. (2019).

Active MS lesions display a predominantly Th1/Th17‐driven immune profile, involving CD4+ and CD8+ T cells, natural killer cells, macrophages, and activated microglia. This cellular activity promotes the release of several pro‐inflammatory cytokines, including IL‐1, IL‐2, IL‐17, IL‐22, TNF‐α, and IFN‐γ. Once activated, T lymphocytes enter the CNS by binding to endothelial surfaces through α4‐integrins (LFA‐1 and VLA‐4), their corresponding receptors (ICAM‐1 and VCAM‐1), and selectins with their ligands. These interactions enable lymphocyte rolling and adhesion on endothelial cells. Their passage across the blood–brain barrier is further enhanced by metalloproteinases such as MMP‐2 and MMP‐9 (Gavrilă et al. 2026).

Within the CNS, activated CD4+ T cells undergo reactivation after recognizing myelin antigens presented via MHC class II molecules. CD8+ T cells, macrophages, and microglia act as key effector cells in the Th1‐type immune response and are commonly found in early MS lesions. It remains unclear whether the resulting loss of myelin and oligodendrocytes arises from nonspecific damage mediated by metalloproteinases, inflammatory cytokines, reactive oxygen species (ROS), and nitric oxide (NO), or whether it reflects a targeted immune attack on myelin or oligodendrocyte antigens intensified by local inflammation (Dargahi et al. 2017; Restuccia et al. 2025). Regardless of the mechanism, these events contribute to cognitive impairment and broader immune alterations.

Anxiety is one of the most frequent neuropsychiatric comorbidities in patients with multiple sclerosis and has been increasingly recognized as an important factor influencing disease burden and quality of life. Recent studies indicate that anxiety symptoms are strongly associated with chronic inflammation, neuroimmune dysregulation, fatigue severity, cognitive dysfunction, and uncertainty related to disease progression (Boeschoten et al. 2017; Le et al. 2024). In addition to psychological stress related to diagnosis and disability, inflammatory cytokines and persistent CNS immune activation may directly contribute to anxiety development by affecting neurotransmission and neural circuits involved in emotional regulation (Gómez‐Melero et al. 2024; De Meo et al. 2026). The neural substrates of anxiety and fatigue in MS involve disruption of specific brain circuits and neuroendocrine systems that regulate mood, motivation, and arousal. Neuroinflammation and cytokine signaling modulate the hypothalamic‐pituitary‐adrenal (HPA) axis—the body's central stress response system. Studies have reported HPA axis hyperactivity in fatigued MS patients, with elevated adrenocorticotropic hormone (ACTH) responses to dexamethasone‐CRH challenge, suggesting that cytokine‐driven dysregulation of neuroendocrine stress pathways may contribute to both fatigue and anxiety symptoms (Gottschalk et al. 2005; Gold et al. 2011). However, findings are heterogeneous across cohorts, with some studies reporting no clear HPA‐fatigue correlation, indicating that HPA involvement may vary by disease subtype, disability level, or individual patient characteristics (Heesen et al. 2003).

Anxiety may also worsen treatment adherence, social functioning, and perception of fatigue, reinforcing its relevance as both a clinical symptom and a potential modifier of disease outcomes. Therefore, investigating anxiety together with inflammatory markers, cognitive impairment, and neuromuscular fatigue may provide a more comprehensive understanding of the multidimensional impact of multiple sclerosis. Collectively, these findings suggest that MS‐related anxiety and fatigue arise not solely from lesion burden or physical disability, but from a complex interplay of neuroinflammation, glial activation, oxidative and mitochondrial dysfunction, and disruption of monoaminergic and neuroendocrine circuits. This mechanistic framework—often termed the “sickness behavior” model—posits that pro‐inflammatory cytokines produce a coordinated set of behavioral changes (fatigue, anhedonia, social withdrawal, and anxiety) that overlap substantially with the neuropsychiatric symptom profile observed in MS (Dantzer et al., 2008; Morris et al., 2018). Understanding these mechanisms is essential for developing targeted interventions that address the biological underpinnings of anxiety and fatigue, rather than treating them as purely psychological or secondary phenomena.

Based on this background, multiple sclerosis should be understood as a multifactorial disease in which genetic susceptibility, chronic neuroinflammation, and environmental factors interact to promote demyelination, neuronal injury, and progressive neurological dysfunction. These mechanisms are closely associated not only with physical disability, but also with cognitive impairment, neuromuscular fatigue, and emotional disturbances such as anxiety, which significantly affect patients’ quality of life. Persistent inflammatory activity and immune dysregulation may contribute simultaneously to fatigue progression, neuronal damage, and neuropsychological symptoms, reinforcing the importance of investigating these outcomes in an integrated manner. Therefore, the aim of the present study was to assess cognitive function, neuronal injury, inflammatory response, anxiety, and their relationship with neuromuscular fatigue in the serum of individuals diagnosed with multiple sclerosis.

## 2 Material and Methods

### 2.1 Study Design

A cross‐sectional case‐control study was conducted. The study was approved by the Research Ethics Committee of the Universidade do Extremo Sul Catarinense (UNESC) under opinion number 5,264,095 and was carried out in accordance with the Declaration of Helsinki.

#### 2.1.1 Setting

This cross‐sectional case‐control study was conducted in a municipality in southern Brazil between February 2022 and May 2023. Participants were recruited according to predefined eligibility criteria. The study included 20 newly diagnosed, treatment‐naïve patients with relapsing‐remitting multiple sclerosis, or RRMS, and 20 healthy controls matched by age and sex. All patients with MS were recruited at the time of diagnosis and before the initiation of any MS‐specific pharmacological treatment, including disease‐modifying therapies, corticosteroids, immunosuppressive therapy, and symptomatic treatments for MS. Patients with primary progressive MS, secondary progressive MS, clinically isolated syndrome, other demyelinating diseases, or other neurological disorders were not enrolled.

#### 2.1.2 Participants

The case group consisted of 20 patients with newly diagnosed RRMS who met the eligibility criteria. The diagnosis of RRMS was established by an experienced neurologist according to the 2017 revised McDonald criteria. Inclusion criteria for the MS group were as follows: diagnosis of RRMS; age between 18 and 50 years; preserved cognitive and communicative capacity to answer the questionnaires; and absence of relevant physical or psychiatric comorbidities that could interfere with clinical or biochemical assessments.

Patients were excluded if they had primary progressive MS, secondary progressive MS, clinically isolated syndrome, other demyelinating diseases, or other neurological disorders. Patients who had received disease‐modifying therapy, corticosteroids, immunosuppressive therapy, anti‐inflammatory treatment, or symptomatic pharmacological treatment related to MS before blood collection were also excluded. The age range of 18–50 years was selected to include adult patients within the typical age range of RRMS, while excluding pediatric‐onset and older‐onset MS, which may differ in clinical trajectory, comorbidity burden, inflammatory profile, and neurodegenerative characteristics.

**FIGURE 1:**
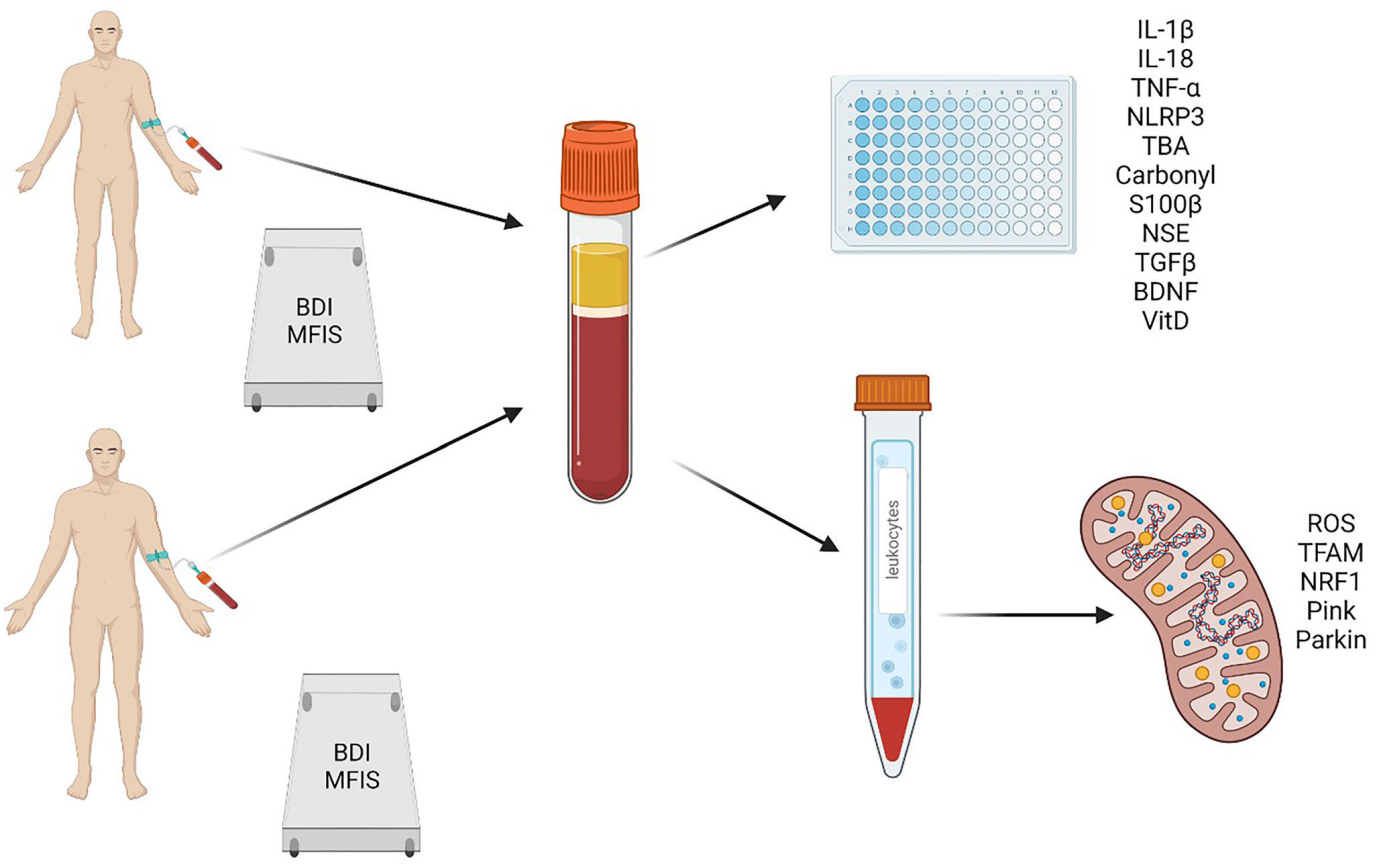
Study Design. Initially, individuals diagnosed with MS were identified and, if they met the inclusion criteria, they were submitted to a sociodemographic questionnaire and to a screening for symptoms of neuromuscular fatigue and anxiety. Soon after, the blood of these individuals was collected for biochemical and immunochemical analysis. According to the group of individuals, an individual matched by gender and age was selected and submitted to the same procedures.

The control group consisted of 20 healthy individuals from the same geographic region, matched to the RRMS group by age and sex. Controls were eligible if they were 18–50 years old, had no diagnosis of MS or other neurological disorders, and reported no chronic medical conditions. Exclusion criteria for controls included a history of neurological disease, autoimmune or chronic inflammatory disorders, acute or chronic infections, psychiatric disorders, diabetes mellitus, uncontrolled thyroid disease, cardiovascular disease, chronic renal or hepatic disease, malignancy, pregnancy, and regular use of anti‐inflammatory drugs, corticosteroids, immunosuppressive agents, psychotropic medications, or neuroactive drugs. These criteria were adopted to minimize potential confounding effects on fatigue, anxiety, inflammatory status, oxidative stress, mitochondrial parameters, neurotrophic factors, and peripheral biomarkers of neuronal injury. The study design and participant allocation are summarized in Figure 1.

### 2.2 Procedures

Investigators monitored the health network daily to identify potential participants, and individuals who met the inclusion criteria were deemed eligible for enrollment. Eligible patients were approached directly and invited to join the study; once informed consent was obtained, data collection was scheduled at the UNESC Pathophysiology Laboratory. At the same time, after confirming cases of RRMS, recruitment of participants for the control group was initiated.

All data were collected during a single visit of no more than one hour. At the start of the session, each participant was assigned an identification code and completed a sociodemographic questionnaire, followed by two validated assessments: the Beck Anxiety Inventory (BAI) and the MFIS. Immediately afterward, 5 mL of venous blood were drawn from the cubital vein using EDTA‐containing tubes. Blood samples were collected in the morning after fasting, before the initiation of any MS‐specific pharmacological treatment. Thus, all biochemical, inflammatory, oxidative stress, mitochondrial, and neuronal injury markers were assessed in treatment‐naïve patients.

The biological samples were processed and fractionated, with serum stored at –80°C for subsequent analyses, including inflammatory markers (TNF‐α, IL‐1β, and IL‐10), indicators of neuronal injury (Enolase and S100β), and neuroplasticity markers (BDNF and vitamin D). The concentrated cellular fraction of the blood was separated, and lymphocytes were isolated for the evaluation of mitochondrial damage.

### 2.3 Beck Anxiety Inventory (BAI)

The BAI, developed by Aaron Beck (1990), is a brief measure of anxiety with a focus on somatic symptoms of anxiety that was developed as a measure adept at discriminating between anxiety and depression. It is a self‐administered questionnaire composed of 21 multiple‐choice items. Each of the 21 items reflects the individual's current state and is rated on a four‐point scale ranging from 0 to 3. The aim of the instrument is to quantify the level of anxiety, and it is recognized for being simple to administer and highly accepted in clinical and research settings. Scoring is easily accomplished by summing scores for items. The total score ranges from 0 to 63. The following guidelines are recommended for the interpretation of scores: 0–9, normal or no anxiety; 10–18, mild to moderate anxiety; 19–29, moderate to severe anxiety; and 30–63, severe anxiety.

### 2.4 Modified Fatigue Impact Scale (MFIS)

The MFIS (Fischer et al. 1999) and the Fatigue Severity Scale (FSS) (Krupp et al. 1989) were originally created as general tools to evaluate fatigue. Over time, they have become the most widely applied instruments for assessing fatigue in individuals with multiple sclerosis (MS). Due to the need for an MS‐specific assessment, both scales were adapted and underwent cross‐cultural validation for use in Brazil. The Brazilian version of the Modified Fatigue Impact Scale (MFIS/BR) (Pavan et al. 2007) distinguishes three key dimensions of fatigue—physical, cognitive, and psychosocial. It consists of 21 items, each rated on a scale from 0 to 4, based on symptoms experienced in the preceding two weeks.

The total MFIS score can range from 0 to 84 and is obtained by summing all item responses, providing an overall indication of how fatigue affects the patient's daily functioning. A score below 38 suggests no significant fatigue, whereas scores of 38 or higher indicate increasing levels of fatigue impact. The Brazilian version of the Fatigue Severity Scale (FSS/BR) contains nine items designed to detect signs of fatigue (Mendes et al. 2020). Each item is scored from 1 to 7, also considering the previous two‐week period. Total scores vary from 9 to 63, with values under 27 indicating no fatigue. Scores from 27 and above reflect fatigue, categorized as mild (28–39), moderate (40–51), or severe (52–63).

For the present study, we selected the MFIS as the primary measure of fatigue, as it is regarded as the most appropriate tool for assessing fatigue in MS. Compared with the FSS and its Brazilian adaptation (FSS/BR), the MFIS demonstrates fewer measurement issues and stronger psychometric properties (Mills et al. 2009).

### 2.5 Immunobiochemical Evaluation

After collection, the samples were sent to the Pathophysiology Laboratory at the University of Extremo Sul Catarinense for processing. After blood collection, samples were allowed to clot and were subsequently centrifuged at 1000 × g for 20 min. The serum fraction was then carefully separated from the cellular components and stored at −80°C until analysis. Prior to biochemical assessments, serum samples were removed from storage approximately one hour before testing to allow complete thawing.

Quantification of IL‐1β (DY201), TNF‐α (DY210), BDNF (DY248), IL‐18 (DY318‐05), NSE (DY5169‐05), S100β (DY1820‐05), and vitamin D (DY3778B‐05) was performed using sandwich ELISA immunoassays, following the protocols provided with the R&D Systems kits. All procedures adhered strictly to the manufacturer's instructions.

For the assay setup, samples were homogenized in 1 mL of 1× phosphate‐buffered saline (PBS). Capture antibodies, diluted in PBS, were dispensed into the wells of the ELISA plate, which was then incubated overnight at 4°C. The following day, wells were washed with a buffer consisting of 0.05% Tween‐20 in PBS (pH 7.2). Plates were then blocked with 200 µL of a solution containing 1% bovine serum albumin in PBS and incubated.

After the blocking step and subsequent washes, 100 µL of each sample or standard—both diluted in standard diluent (0.05% Tween‐20 and 0.1% BSA in PBS, pH 7.2)—were added to the wells. Plates were sealed and incubated overnight once more. On the next day, the wells were washed again, and 100 µL of the appropriate detection antibodies (IL‐1β, TNF‐α, BDNF, IL‐10, enolase, S100β, or vitamin D), previously diluted in the same diluent, were added. Plates were then incubated for two hours at room temperature.

Following another series of washes, 100 µL of streptavidin–peroxidase polymer solution was dispensed into each well, and the plates were covered with aluminum foil and incubated for 30 min. After this step, wells were washed five times, and 100 µL of tetramethylbenzidine (TMB) substrate was added, allowing color development for 30 min at room temperature. The reaction was stopped by applying 50 µL of 2N hydrochloric acid to each well. The optical density was then measured at 450 nm using a spectrophotometer.

### 2.6 Thiobarbituric Acid Reactive Species Levels

Lipid oxidative damage was evaluated through the quantification of TBARS produced during an acid‐induced heating reaction, following the general procedure described by Draper and Hadley (1990). For analysis, each sample was combined with 1 mL of 10% trichloroacetic acid and 1 mL of 0.67% thiobarbituric acid, then incubated under heat for 30 min. The resulting TBARS concentration was measured spectrophotometrically by reading absorbance at 532 nm.

### 2.7 Protein Carbonyl Levels

Protein oxidative damage was evaluated by quantifying carbonyl group formation using the dinitrophenylhydrazine (DNPH) reaction, following the method described by Levine et al. (1990). Proteins were first precipitated with 20% trichloroacetic acid and then resuspended in DNPH solution. The carbonyl content was determined by measuring absorbance at 370 nm with a spectrophotometer.

### 2.8 Protein Determination

Oxidative damage parameters and cytokine levels were normalized to the protein content according to Levine et al. (1990) and Lowry et al. (1951).

### 2.9 Sample Collection and Cell Isolation

Approximately 5 mL of peripheral blood was collected by venipuncture into heparinized tubes from both MS patients and control participants. All samples were obtained in the morning, between 8:00 and 10:00 a.m., with individuals in a fasting state. Peripheral blood mononuclear cells (PBMCs) were separated using Ficoll‐Paque (GE Healthcare, USA) density‐gradient centrifugation following the manufacturer's protocol.

The isolated PBMC layer—composed primarily of lymphocytes—was washed with phosphate‐buffered saline (PBS) and centrifuged at 1300 RPM for 10 min. After discarding the supernatant, the pellet was resuspended in PBS, and this washing step was repeated twice. A final centrifugation at 1200 RPM for 5 min was performed, the supernatant was removed, and the resulting pellet was stored at −80°C for subsequent analyses (Bøyum 1976).

### 2.10 Western Blotting

Levels of NRLP3, Bax, and Bcl‐2 proteins were assessed by Western blotting using protein extracts obtained from isolated blood cells. In summary, samples were lysed in Laemmli buffer (62.5 mM Tris–HCl, pH 6.8, 1% SDS, and 10% glycerol), and 30 µg of protein per lane were separated by SDS–PAGE, followed by transfer onto nitrocellulose membranes. Successful protein transfer and loading were confirmed with Ponceau S staining. Membranes were then blocked in Tween–Tris‐buffered saline (TTBS; 100 mM Tris–HCl, pH 7.5, 0.9% NaCl, 0.1% Tween‐20) supplemented with 5% albumin.

Following blocking, membranes were incubated overnight at 4°C with primary antibodies diluted 1:1000 in TTBS (NRLP3, Abcam ab214185; Bax, Abcam ab32503; Bcl‐2, and Abcam ab194583). After washing with TTBS, membranes were incubated for 2 h at room temperature with species‐appropriate HRP‐conjugated secondary antibodies (Abcam: ab6789mg1) (1:5000). Immunoreactive bands were visualized using an enhanced chemiluminescence detection system (ECL). Band intensities were quantified using ImageJ software, ensuring that all blots were developed within the linear detection range for densitometric analysis.

### 2.11 Mitochondrial Isolation

Blood‐derived cell fractions were homogenized in a buffer composed of 225 mM mannitol, 75 mM sucrose, 1 mM EGTA, 0.1% bovine serum albumin, and 10 mM HEPES, adjusted to pH 7.2. The resulting homogenate was first centrifuged at 2000 × g for 3 min at 4°C. The supernatant from this step was subsequently centrifuged at 12,000 × g for 8 min at 4°C. The resulting pellet was then resuspended in 12mL of isolation buffer supplemented with 20 µL of 10% digitonin and centrifuged again at 12,000 × g for 10 min at 4°C.

Afterward, the pellet was washed by resuspension in isolation buffer without EGTA and subjected to another centrifugation at 12,000 × g for 10 min at 4°C. The final mitochondrial fraction was resuspended in EGTA‐free isolation buffer to achieve an approximate protein concentration of 20 mg/mL. Once isolated, the mitochondrial samples were rapidly frozen at −80°C until further analyses could be performed.

### 2.12 ROS Levels in Mitochondria

Intramitochondrial ROS generation was assessed using the non‐fluorescent, cell‐permeable probe 2',7'‐dichlorofluorescein diacetate (DCFH‐DA). Once inside the mitochondria, intracellular esterases converted DCFH‐DA into dichlorofluorescein (DCFH), a non‐fluorescent intermediate that becomes fluorescent dichlorofluorescein (DCF) following oxidation by reactive oxygen species. Mitochondrial preparations were incubated with 10 µM DCFH‐DA for 30 min at 37°C. Following incubation, samples were rinsed with PBS containing 0.2% Triton X‐100. Fluorescence was then quantified using a microplate reader (SpectraMax GEMINI XPS, Molecular Devices, USA) with excitation at 485 nm and emission at 520 nm. Results were normalized and expressed as fluorescence units per microgram of protein.

### 2.13 Analysis of 8‐oxo‐G Levels

Mitochondrial DNA (mtDNA) was isolated from blood‐derived cell fractions. Both extraction and quantification were carried out using commercial kits supplied by Abcam. Samples were first homogenized in Cytosol Extraction Buffer, followed by the addition of Mitochondrial Lysis Buffer and ethanol. The resulting mtDNA fraction was then resuspended and purified for subsequent 8‐oxoG analysis.

To measure 8‐oxoG levels, a sandwich ELISA assay was performed using the Biolabs Cell Kit, following the manufacturer's instructions. The reaction was terminated by adding 100 µL of stop solution, and absorbance was recorded at 450 nm using a spectrophotometer.

### 2.14 RT—PCR Analysis

Total RNA was extracted using the Trizol reagent (Invitrogen, USA) according to the manufacturer's protocol. To prevent contamination by genomic DNA, RNA purity was assessed by spectrophotometry (A260/280 nm), after which the samples were treated with Deoxyribonuclease I (Invitrogen). Complementary DNA (cDNA) was synthesized using the ImProm‐II Reverse Transcription System, following the supplier's instructions.

Quantitative PCR was carried out with SYBR Green (Invitrogen) on a 7500 Fast Real‐Time System (Software v.2.0.5, Applied Biosystems). The primer sequences for TFAM, NRF‐1, Parkin, and Pink‐1 are provided in Supplementary Material—Figure S1.

Previous work from our laboratory showed differential expression of these genes when comparing Sham and CLP animals (Michels et al. 2020). Therefore, in alignment with the 3Rs principle, a sham group was not included in the present gene expression analysis, and comparisons were restricted to treated versus untreated CLP animals.

### 2.15 Statistical Analysis

For the construction of the database and statistical calculations, the statistical programs Statistical Package for the Social Sciences 22 (SPSS) and GraphPad Prism 9.0 were used. The results of continuous variables are presented as mean ± standard deviation, and comparisons between groups (control and MS) were performed using Student's *t* test for paired samples. *p*‐values < 0.05 were considered statistically significant. Correlation analyses were conducted to explore associations among clinical and biochemical variables. All possible pairwise correlations among numerical variables were calculated. Spearman's rank correlation coefficient was used as the primary correlation method because of the small sample size and the non‐normal distribution of several biomarkers. Correlations were classified according to the absolute Spearman's rho value as weak when 0.20≤∣rho∣<0.400.20≤∣rho∣<0.40, moderate when 0.40≤∣rho∣<0.600.40≤∣rho∣<0.60, strong when 0.60≤∣rho∣<0.800.60≤∣rho∣<0.80, and very strong when ∣rho∣≥0.80∣rho∣≥0.80. The complete correlation table and heatmap were provided as supplementary material.

## 3 Results

**FIGURE 2:**
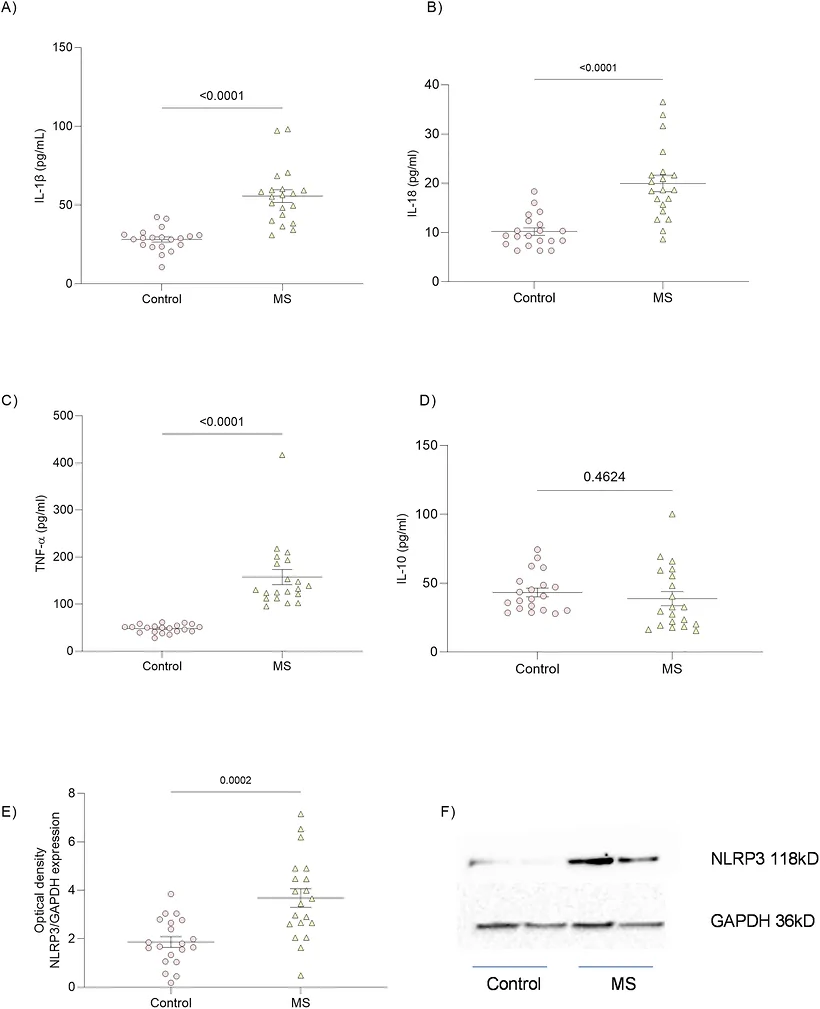
MS patients have increased levels of pro‐inflammatory cytokines and high expression of the NLRP3 inflammasome in serum. Blood of patients with MS were collected and the serum isolated for the determination of (A) IL‐1β, (B) IL‐18, (C) TNF‐α levels, and (D) NLRP3 expression. Data are presented as mean ± SEM, with the statistical difference calculated using the student *t* test for paired samples. *p < 0.05 vs. control group. *n* = 20.

In the MS group, all 20 participants had relapsing‐remitting multiple sclerosis, RRMS. All of these subjects were matched for sex and age with healthy subjects. The sample is aged between 18 and 50 years, 50% male and 50% female; 66.6% of the sample is white and has completed higher education (20.8%), as per Supplementary Material—Figure 2. It is assumed that all individuals only have a diagnosis of RRMS, with no other comorbidities. 17 individuals with MS had high levels of severe anxiety; moreover, when evaluating fatigue, it was observed that 19 individuals had symptoms of neuromuscular fatigue, as shown in Table 1. As for the control group, 20 patients had no fatigue; that is, 100% of the control group did not show fatigue.

**TABLE 1:** Higher incidence of neuromuscular fatigue and anxiety in MS patients compared to healthy individuals. Table 1 shows the mean ± SEM of the clinical scores of the MFIS and BDI tests. The statistical difference calculated using the student *t* test for paired samples. *p < 0.05 vs. control group.

Scores	Control		SM	Sig
**Beck Anxiety Inventory**				
0‐10 (minimal anxiety)	9 (45%)		1 (5%)	0,0042
11‐19 (anxiety mild)	1 (5%)		—	
20‐30 (anxiety moderate)	7 (35%)		2 (10%)	
31‐63 (anxiety severe)	3 (15%)		17 (85%)	
**Scale MIFS**				
Absence of neuromuscular fatigue		20 (100%)	1 (5%)	0,0012
Presence of neuromuscular fatigue		—	19 (95%)	

In serum from individuals with MS, increased levels of pro‐inflammatory cytokines such as IL1β (Figure 2A), IL18 (Figure 2B), and TNFα (Figure 2C) were found compared to the control group (*p* < 0.05). However, IL‐10, which has anti‐inflammatory actions, this alteration was not observed (Figure 2D). Interestingly, with the increase in IL1β and IL18, the activation of the NLRP3 inflammasome was analyzed (Figure 2E), which was increasing its expression in MS patients. At the same time, this increase may be related to the cleavage and maturation of proinflammatory cytokines, characterizing a possible chronic inflammation.

**FIGURE 3:**
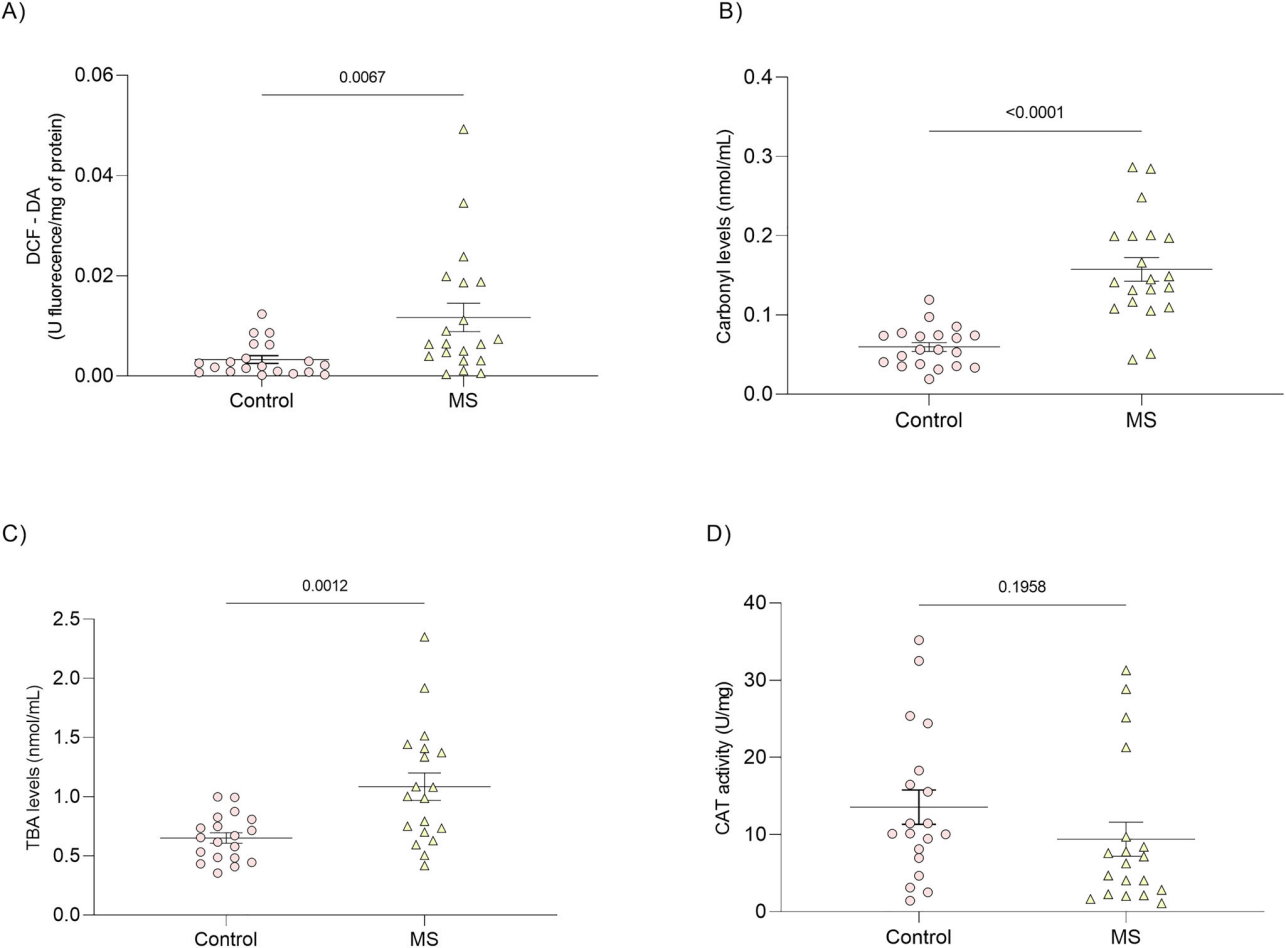
Increased production of mitochondrial ROS and increased oxidative damage is found in MS patients. Blood of patients with MS were collected and the serum isolated for the determination of (A) DCF‐DA, (B) Carbonyl, (C) TBA, and (D) CAT levels. Data are presented as mean ± SEM, with the statistical difference calculated using the student *t* test for paired samples. *p < 0.05 vs. control group. *n* = 20.

**FIGURE 4:**
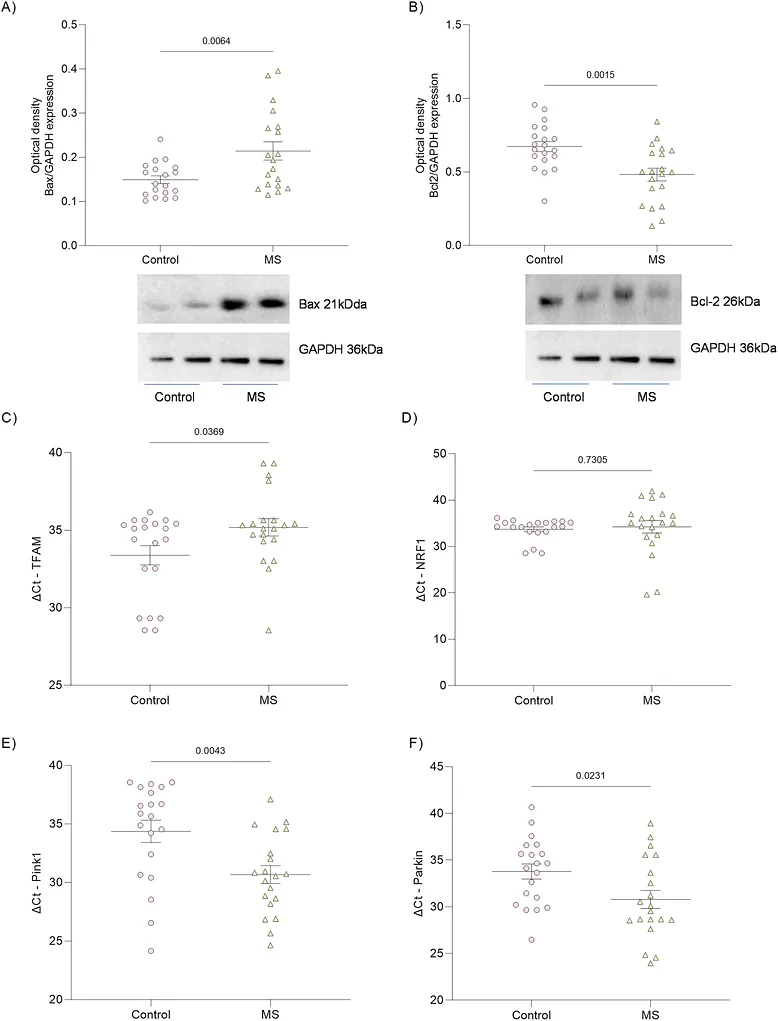
MS patients showed increased expression of pro‐apoptotic protein and mitophagy and decreased mitochondrial biogenesis in isolated lymphocyte mitochondria. Blood of patients with MS were collected and the isolated lymphocyte mitochondria for the determination of (A) Bax/GAPDH, (B) Bax/GAPDH, (C) TFAM, (D) NRF1, (E) Pink1, and (F) Parkin expression. A and B protein expression in relation to GAPDH. C, D, E and F gene expression was quantified by the ∆Ct. Data are presented as mean ± SEM, with the statistical difference calculated using the student *t* test for paired samples. *p < 0.05 vs. control group. *n* = 20.

In fact, increased oxidative damage was observed in patients with RRMS, as evidenced by higher levels of protein oxidation markers and lipid peroxidation (Figures 3B and 3C, respectively) in serum samples compared with healthy controls. In addition, mitochondria isolated from leukocytes exhibited increased ROS production and higher 8‐oxoG levels (Figure 3A), indicating enhanced mitochondrial oxidative damage. Catalase (CAT) activity, assessed as a marker of antioxidant enzymatic defense, did not differ significantly between RRMS patients and healthy controls (Figure 3D). Therefore, despite the increased oxidative damage observed, CAT activity remained relatively preserved, suggesting that this antioxidant enzymatic defense mechanism was not significantly altered in the evaluated patients. With the increase in ROS production by mitochondria isolated from leukocytes, Bax and Bcl‐2 proteins (Figures 4A and 4B) were analyzed, and it was observed that individuals with MS have an increase in pro‐apoptotic proteins and a decrease in anti‐apoptotic proteins. In addition, patients showed a decrease in the expression of TFAM genes (Figure 4C), responsible for mitochondrial biogenesis and an increase in Parkin and Pink‐1 expression (Figures 4E and 4F), that are related to mitophagy processes when compared to the control group. It is noteworthy that the amplification of NRF1 (Figure 4D), of both groups did not differ.

**FIGURE 5:**
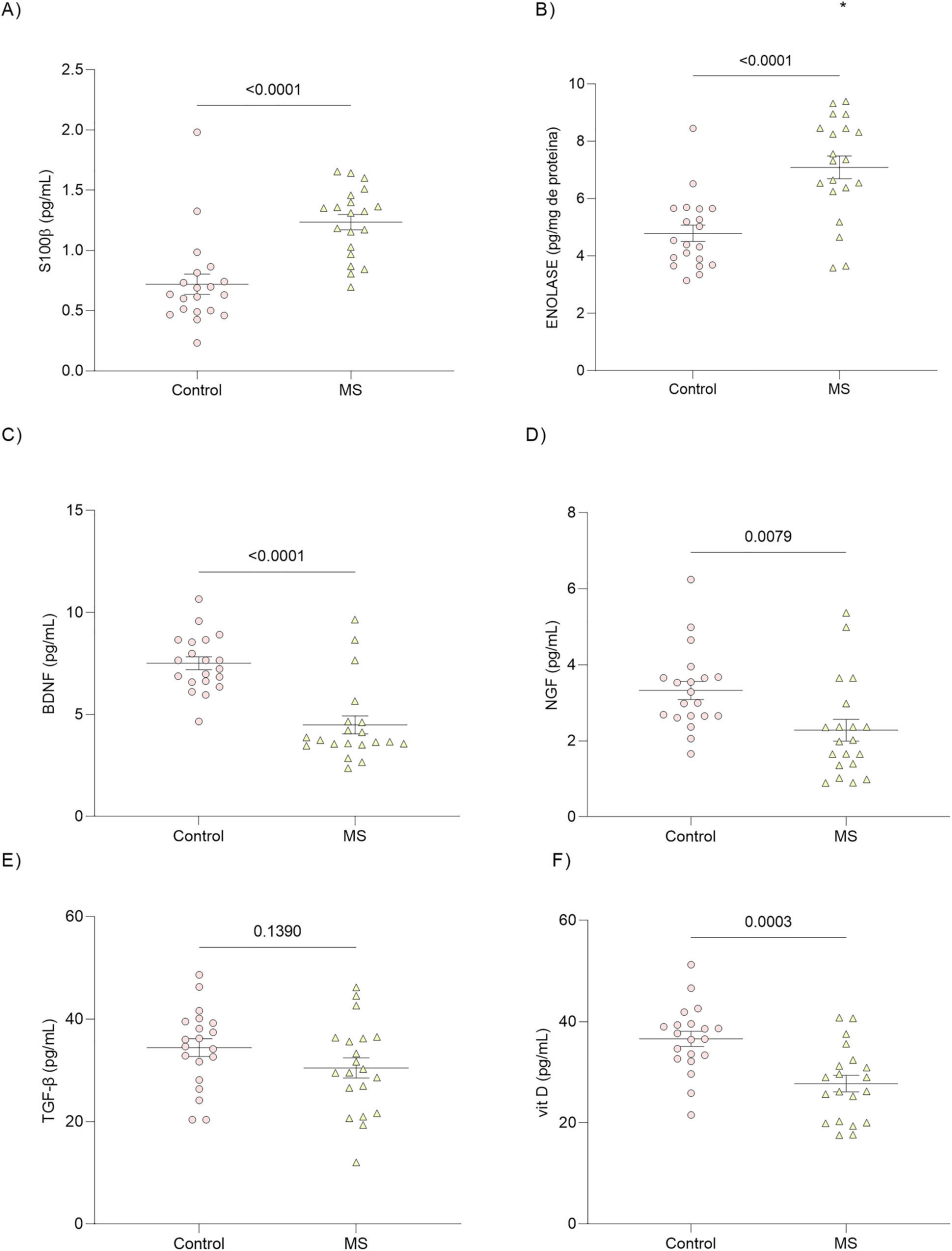
Increase S100β NSE and Vit D and decrease in BDNF and NGF in serum from MS patients. Blood of patients with MS were collected and the serum isolated for the determination of (A) S100β, (B) NSE, (C) BDNF, (D) NGF, (E) TGF‐β, and (F) Vitamin D levels. Data are presented as mean ± SEM, with the statistical difference calculated using the student *t* test for paired samples. *p < 0.05 vs. control group. *n* = 20.

With this process of mitochondrial damage installed, increased ROS which in turn increases NLRP3 activity and consequent maturation of pro‐inflammatory cytokines, the levels of biomarkers of neuronal injury were analyzed in patient serum and an increase in S100β and NSE (Figures 5A and 5B) was observed. Neuronal activity and the decrease of neurotrophins such as BDNF and NGF (Figures 5C and 5D) in patients with MS. It should be noted that no changes were found in TGF‐β levels (Figure 5E), but a decrease in vitamin D (Figure 5F) was observed in these individuals.

**FIGURE 6:**
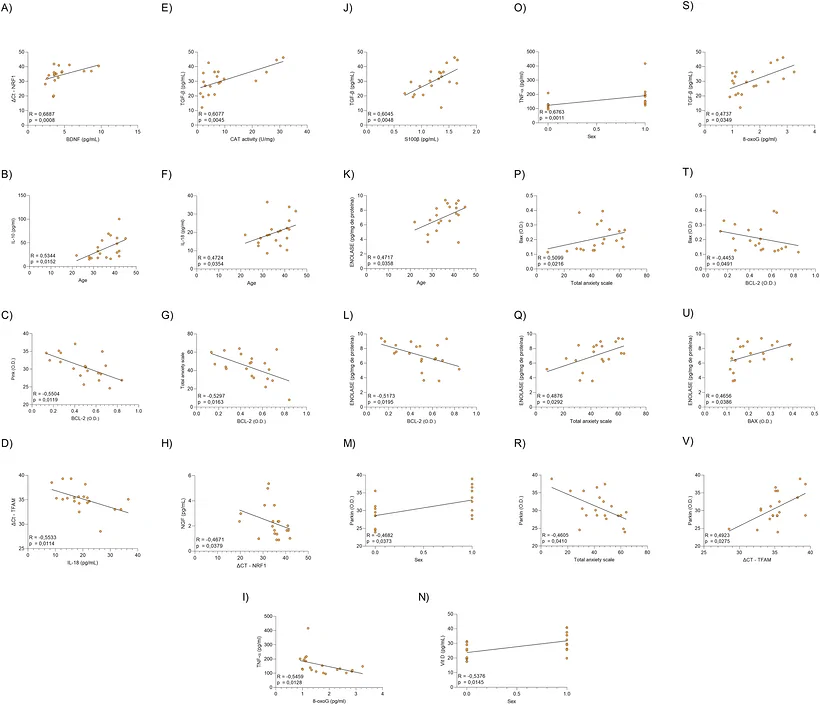
Key correlations among inflammatory, mitochondrial, oxidative, neurotrophic, apoptotic, and mitophagy markers in treatment‐naïve RRMS patients. All possible pairwise correlations among numerical variables were calculated. Spearman's rho and raw p‐values are shown. *n* = 20. (A) Positive correlation between BDNF and ΔCt‐NRF1; (B) Positive correlation between age and IL‐10 levels; (C) Negative correlation between BCL‐2 and Pink levels; (D) Negative correlation between IL‐18 and ΔCt‐TFAM; (E) Positive correlation between CAT activity and TGF‐β levels; (F) Positive correlation between age and IL‐18 levels; (G) Negative correlation between BCL‐2 and total anxiety scale score; (H) Negative correlation between ΔCt‐NRF1 and NGF levels; (I) Negative correlation between 8‐oxoG and TNF‐α levels; (J) Positive correlation between S100β and TGF‐β levels; (K) Positive correlation between age and Enolase levels; (L) Negative correlation between BCL‐2 and Enolase levels; (M) Positive association between sex and Parkin levels; (N) Positive association between sex and Vit D levels; (O) Positive association between sex and TNF‐α levels; (P) Positive correlation between total anxiety scale score and Bax levels; (Q) Positive correlation between Total Anxiety Scale score and Enolase levels; (R) Negative correlation between Total Anxiety Scale score and Parkin levels; (S) Positive correlation between 8‐oxoG and TGF‐β levels; (T) Negative correlation between BCL‐2 and Bax levels; (U) Positive correlation between Bax and Enolase levels; and (V) Positive correlation between ΔCt‐TFAM and Parkin levels.

Exploratory correlation analyses were performed to investigate the relationships among clinical and biochemical variables in untreated patients with multiple sclerosis (Figure 6). The strongest biochemical correlation was observed between BDNF and NRF1 (Spearman's rho = 0.689, *p* = 0.000785), suggesting a potential relationship between neurotrophic signaling and mitochondrial biogenesis. Catalase was positively correlated with TGF‐B (rho = 0.608, *p* = 0.004476), and S100 was also positively correlated with TGF‐B (rho = 0.605, *p* = 0.004753), indicating possible links between immunoregulatory signaling, antioxidant defense and neural/glial‐related markers.

Regarding the inflammatory‐mitochondrial axis, IL‐18 was negatively correlated with TFAM (rho = −0.553, p = 0.011379), suggesting that higher mitochondrial DNA maintenance may be associated with lower inflammatory activity. In addition, BCL‐2 was negatively correlated with Pink (rho = ‐0.550, p = 0.011920), indicating a possible relationship between apoptotic regulation and mitophagy‐related pathways. The complete correlation table and Spearman correlation matrix were included as Supplementary Figures S3 and S4.

## 4 Discussion

Observed that patients with MS have high levels of anxiety and muscle fatigue assessed by the Beck et al. (1988) scale, which, in turn, is related to the progression of the disease, as demonstrated in the studies by Eftekhari et al. (2012) and Morrow (2018) who described fatigue as a particularly common symptom in MS patients, affecting up to 85% of patients, leading to decreased mobility, which leads to impairment of functional capacity and, consequently, causes negative effects on the quality of life. These changes in quality of life are directly related to the evolution of the disease, impacting the mental health of the bearer, or rather, the development of severe clinical conditions of anxiety and worsening (Jiménez‐Zambrano, Zambrano‐Llaguno, and Acuña‐Chong 2020; Howard et al., 2020; Panda et al. 2018).

Corroborating with the present study, Julian and Arnett (2009) demonstrated that neuromuscular fatigue has a great impact on the life of a person with MS and may lead to the development of anxiety. Furthermore, a study by Reese et al. (2013) showed that over the years, MS can develop new symptoms related to physical and mental changes that may be related to the inflammation caused by the disease. The study by STOUT and FINLAYSON also indicated a greater difficulty for people with MS in usual activities such as leisure and activities of daily living, generating functional dependence to carry out their activities.

As the process of demyelination makes it more difficult for neurons to send signals, it is necessary to increase neuronal activity in cellular tissues to compensate for this deficit, which gradually leads to weakness of physical capacities and results in the depletion of physical components in MS (Eftekhari et al. 2012). Furthermore, it is believed that neuromuscular fatigue and anxiety may be related to the inflammatory process, which in turn is related to the increased levels of IL‐1β and TNFα levels observed in the present study.

TNFα is elevated in active lesions in the serum and cerebrospinal fluid (CSF) of patients with MS and is correlated with the neuroinflammatory process, neuronal apoptosis, death of glial cells, and cognitive damage, with consequent disease progression.(Hemmerich et al. 2022). The increase in this cytokine is related to the activation of intracellular cascades that lead to the degeneration of oligodendrocytes by necroptosis, with consequent demyelination (Ofengeim et al. 2015; Zveik et al. 2022). Furthermore, the inflammatory process caused by pro‐inflammatory cytokines with IL‐1β and 18 induces the activation of macrophages and microglia, which, in turn, produces increased microglial activation (increase of Iba markers), exacerbation of neuroinflammation, and production of ROS and nitric oxide, culminating in demyelination and axonal loss (Panda et al. 2018; Jiménez‐Zambrano et al. 2020; Hemmerich et al. 2022; Zveik et al. 2022).

NLRP3 is a key mediator in the progression of MS, given the cleavage and maturation of these cytokines that induce the inflammatory process; however, the exact mechanism is still unclear (Shao et al. 2021; Gris et al. 2010). It is known that since these autoinflammatory diseases are often associated with gain‐of‐function mutations in the human *NLRP3* gene, it is possible that the NLRP3 inflammasome is also overactive in MS (Gris et al. 2010; Inoue and Shinohara 2013). This hypothesis can be supported due to the increase in IL‐1β and IL‐18 found in the present study, in addition to the increased expression of this inflammasome. In addition, a study with an animal model of autoimmune encephalopathy found high levels of NLRP3, ASC, activated caspase‐1, mature IL‐1β, and IL‐18 in brain and spinal cord tissues (Furlan et al. 1999). Another study showed that NLRP3 inflammasome‐triggered secretion of IL‐1β and IL‐18 in stimulated macrophages increased the expression of chemotaxis‐related proteins and activated CD4+ T cells (Scheu et al. 2019; Ko, Hynecek, and Homa 1979).

With the maturation of IL‐1β and IL‐18 caused by NLRP3, it can generate the differentiation of CD4^+^ T cells for Th1 and Th17 phenotypes, which express a high level of IL‐1R contributing to the pathophysiology and clinical development of MS (Kothari et al. 2018). In addition, studies in mice deficient in the IL‐18 receptor are resistant to autoimmune encephalopathy due to resistance in Th1/Th17 autoreactivity and in promoting Th1 differentiation to induce IFN‐γ (Shi et al. 2000; Gris et al. 2010).

It should be noted that for the activation of NLRP3, a stimulus that is related to the production of intracellular ROS, arising from mitochondrial activity and dysfunction (Elliott and Sutterwala 2015) is fundamental. With the process of mitochondrial dysfunction caused by the neuroinflammatory process, changes occur in the flow of calcium, which is released from the endoplasmic reticulum, causing depolarization of the mitochondrial membrane, stimulating the generation of ROS and exacerbating oxidative damage to lipids and proteins, such as observed in the present study (Shenker et al. 2015); (Horng 2014).

Beyond direct tissue damage, NLRP3 activation has profound implications for the non‐motor symptomatology of MS, specifically fatigue and anxiety. This process is mediated by mitochondrial dysfunction and the increased production of ROS, which act as both triggers and consequences of inflammasome activation (Morris et al. 2018; Malhotra et al. 2020). The resulting microglial activation and sustained cytokine release in the CNS promote a neuroinflammatory state aligned with the “sickness behavior” model. Within this framework, systemic inflammation communicates with the brain to alter neural circuits and the HPA axis, clinically manifesting as neuromuscular fatigue and emotional dysregulation, such as anxiety (Morris et al. 2018).

Mitochondrial damage and subsequent ROS production are closely related to NLRP3 inflammasome activation, and this could influence the pathophysiological mechanisms of MS. In fact, in the present study, an increase in the production of ROS by the mitochondria and consequent lipid and protein damage in individuals with MS was observed; it is emphasized that these alterations may influence the mechanisms of mitochondrial biogenesis or autophagy. In addition, mitochondrial DNA (mtDNA) released from damaged mitochondria functions as a DAMP that can directly activate inflammasomes, increasing the intensity of the inflammatory process (Petersen et al. 1978; Scheller et al. 1975; Kurihara, Narita, Kawaradani, Kikkawa, and Usami 1979).

It is noteworthy that patients with MS showed increased expression of Bax proteins and mitophagy‐inducing factors (Yao et al. 2021) (Pink and Parkin), while a retrospective study in individuals with MS reported a strong humoral response mediated by them (Yao et al. 2021; Patergnani et al. 2018). Furthermore, increased levels of PARKIN in serum and cerebrospinal fluid (CSF) were found in individuals with MS, indicating that this possible increase may be related to active phases of the disease (Castellazzi et al. 2019). In addition, PINK1‐PARKIN molecules function together in the mitochondrial quality control pathway, and an accumulation may be related to neuroaxonal damage and increased 8‐oxo‐G in mtDNA (Hokari et al. 2016; Cossu et al. 2021).

Mitochondrial dysfunction is considered one of several causes of axonal neuron degradation in MS (Mao and Reddy 2010). In the present study, we show that there is an imbalance causing the decrease in mitochondrial biogenesis (low TFAM and NRF1 expression) related to the possible increase in mythophagy and ROS in blood lymphocytes in relation to healthy controls. This raises the pathophysiological hypothesis that may be related to the deficient mitochondrial biogenesis process (Campbell et al. 2012). There are several connections between and mitochondrial disease, especially Leber's hereditary optic neuropathy. It has been shown that MS is more common and more aggressive in patients with mitochondrial LHON mutations (HARDING et al. 1992; Pfeffer, Burke, Yu‐Wai‐Man, Compston, and Chinnery 2013).

In addition, the clinical severity of mutations in carriers of LHON is related to a defect in mitochondrial proliferation; that is individuals with a greater ability to perform mitochondrial biogenesis are more protected (Giordano et al. 2014; Rojas et al. 2023). Joining the data of these studies, we could formulate the hypothesis that MS is a disease of defective mitochondrial biogenesis. In addition, increased severity in patients with LHON mutations is the result of changes in biogenesis, taking the hypothesis of the importance of maintenance of TFAM/NRF1 to decrease progression speed and ROS.

Other studies have demonstrated that the number of copies of mtDNA and gene expression are deficient in blood samples of patients with MS, suggesting that the number of mtDNA copies and mitochondrial gene expression can be used as a biomarker of the clinical gravity of MS and treatment response (García‐Ruiz et al. 2013; Hayashi et al. 2017; Bhat et al. 2015).

Demyelination results not only in a loss of support by the axons but also in a redistribution of ion channels, destabilizing the axonal membrane potential, reducing excitability, and blocking nerve conduction (Baldassarro, Stanzani, Giardino, Calzà, and Lorenzini 2022). The loss of the myelin sheath in MS causes the Na^+^ channels, normally located in Ranvier's nodes, to be distributed along the axon, changing the type of conduction from saltatory to continuous propagation, with a consequent increase in energy consumption and increase in mitochondria activity, causing an increase in ROS that may be related to the increase in mitophagy and activation of the NLRP3 pathway observed in the present study (Campbell et al. 2012; Baldassarro et al. 2022). Shortly thereafter, under conditions of reduced ATP production, the Na^+^/K^+^ATPase pump, which is normally responsible for exchanging Na^+^ for extracellular K^+^, fails, contributing to an increase in intracellular Na^+^. As a consequence, the Na^+^/Ca^+2^ exchanger, which normally exchanges axoplasmic Ca^2+^ for extracellular Na^+^, begins to function in reverse mode, leading to an increase in Ca^+2^ within the axon and subsequent axonal degenerative responses, which may also trigger the activation of the axon. NLRP3 (Baldassarro et al. 2022; Mahad et al. 2008).

Excessive accumulation of axoplasmic Ca2^+^ leads to the vicious cycle of propagation of mitochondrial dysfunction, decreased energy production, and impairment of axonal transport, culminating in oxidative damage, initiation of the inflammatory process, and changes in glycolytic pathways, increasing NSE levels, observed in this study, in addition to the potent activation of NLRP3 (Mahad et al. 2008; Dias‐Carvalho et al. 2022). In addition, these changes caused in cellular physiology are directly linked to neuromuscular fatigue and anxiety symptoms (Campbell et al. 2012; Dias‐Carvalho et al. 2022).

According to studies Jiménez‐Zambrano et al. (2020), the inflammatory process generated by fatigue and MS can influence neuroendocrine functions, inducing behavioral changes through the modulation of the synthesis of serotonin, dopamine, and melatonin, increasing the number of MS patients with anxiogenic symptoms (Jiménez‐Zambrano et al. 2020). It is also understood that the inflammation observed in the present study may be related to neurodegeneration, mainly with the increase of markers such as S100β and NSE. NSE, which in turn is secreted into the extracellular space after substantial neuronal damage, is linked to the neurodegenerative component of MS (Isgrò et al. 2015).

High levels of NSE are observed at the beginning of the disease; however, with the progression of the disease, especially in the more advanced stages of the same, there is a decrease in production that is probably related to low metabolic activity and great neuronal loss (Håkansson et al. 2017; Dichev, Kazakova, and Sarafian 2020). Thus, it is assumed that NSE autoreactivity is high at the beginning of the disease because the axonal/glial load is still high, as observed in the present study. However, with time, self‐reactivity decreases as neuronal and glial mass decreases, with a consequent decrease in NSE release (Forooghian et al. 2007; Koch et al. 2015).

Under stress conditions, including inflammation of nervous tissue, astrocytes secrete S100β, which has a neurotoxic effect, behaving as a molecular pattern molecule associated with damage. Its increase is known to be related to neurodegenerative diseases, leading to activation of the receptor for advanced glycation end products (RAGE), which acts as a pro‐inflammatory intermediary and inducer of oxidative stress, and may be directly related to inflammasome activity (Donato et al. 2009).

It is known that S100β, when increased, leads to the release of cytokines with IL‐1β and TNF‐α, which may cause apoptosis in neuronal cells and oligodendrocytes due to energy collapse and damage to the mitochondrial membrane, resulting in neuromotor fatigue due to failure in synaptic conduction (Donato et al. 2013; Paknejad, Shirkhanloo, and Aliomrani 2019; De Angelis et al., 2018).

Modulation of S100β levels can be a future treatment for patients with MS, as, according to the study by Di Sante et al. (2020), using animal models of MS demonstrated that the inhibition of S100β was able to modify the neuropathology of the disease, reducing immune infiltrates, decreasing the production of cytokines and ROS, and partially protecting the brain from the damage produced by MS, thus improving the animal behavior.

Furthermore, the reduced availability of BDNF from immune cells and the resulting loss of neuroprotective effects may contribute to neuroaxonal degeneration and loss of neurons in MS (Azoulay et al. 2008; Naegelin et al., 2020). Decreased levels of BDNF may be related to loss of neuroplasticity with consequent changes in neuronal inflammation, memory loss, and neuromuscular changes as well as the development of fatigue (Naegelin et al. 2020). Indeed, BDNF plays important roles in neural survival and synaptic plasticity, but its role in autoimmune diseases such as MS is just beginning to be understood, especially in the development of neuron and oligodendrocyte maintenance and repair (Frota et al. 2009; Chitnis and Weiner 2017).

Studies suggest that reduced BDNF levels are related to reduced tissue protection or, alternatively, that there is an increased consumption of BDNF by the CNS due to tissue damage in MS (Huang and Dreyfus 2016; Dalgas 2017; Nociti, 2020; Shobeiri et al. 2022). Reports on changes in BDNF levels in MS patients are controversial, but in general, BDNF is usually increased during relapse, with normal levels during remission phases. When the process of demyelination occurs, BDNF tends to increase, probably as a compensatory mechanism against neuronal and glial damage (Huang and Dreyfus 2016; Dalgas 2017; Nociti, 2020).

According to a study by Frota et al. (2009), BDNF may play a role in protecting or regenerating brain tissue in patients with MS, so a therapeutic approach that would increase its production may help to slow down or even stop axonal loss (Frota et al. 2009). In fact, a study by Shobeiri et al. (2022), demonstrated that there is some evidence suggesting that exercise can reduce the progression of MS, since intense exercise showed reductions in the development of MS from the increase in peripheral BDNF levels. However, a study by Khademosharie et al. (2018), reported the beneficial effect on the progression of MS in women with 12 weeks of resistance training, and this effect is related to the increase in serum levels of BDNF and vitamin D.

The present study has some limitations that should be acknowledged. First, the relatively small sample size (*n* = 20 patients) may have reduced the statistical power and limited the generalizability of the findings. In addition, although multiple biological pathways related to inflammation, oxidative stress, mitochondrial homeostasis, neurotrophic support, and neuronal/glial injury were evaluated, the present analyses provide an exploratory assessment of these alterations in treatment‐naïve RRMS patients. Therefore, the observed associations should be interpreted as preliminary and hypothesis‐generating rather than conclusive evidence of the underlying pathophysiological mechanisms. Furthermore, the cross‐sectional design precludes causal inferences regarding the relationships among the investigated biomarkers and clinical manifestations. Another limitation is that cerebrospinal fluid (CSF) analyses were not performed, which may have limited the assessment of neuroinflammation and neuronal injury within the central nervous system. CSF samples were not collected because the study involved living patients in whom lumbar puncture was not clinically indicated, and the procedure is invasive and associated with ethical and practical constraints. Future studies involving larger and longitudinal cohorts, as well as the integration of paired serum and CSF biomarkers, are warranted to validate and extend these findings and to provide a more comprehensive understanding of central nervous system involvement in this condition.

## 5 Conclusions

In conclusion, indicators of mitochondrial biogenesis appear to hold promise as potential biomarkers for MS. Further research is needed to clarify how changes in mitochondrial biogenesis correlate with clinical severity, age at onset, and therapeutic outcomes. Additionally, the role of NLRP3 is particularly important, as its strong inflammatory potential may contribute to persistent inflammation. This activity is likely linked to increased oxidative stress and elevated ROS production arising from dysfunctional mitochondria in MS patients, ultimately reducing neuronal integrity and contributing to cognitive impairment and neuromuscular fatigue.

## Author Contributions

A. V. S, F. D. P., T. B., A. M. and D. D. performed the study design, experimental planning and execution of the research and the development of the manuscript. K. D. F., L. F. S. R., F. M. L., M. M. F. M., D. P. B., and L. B. C. helped during the experimental process.

## Funding

This work was supported by Universidade do Extremo Sul Catarinense (UNESC) and Fundação de Amparo à Pesquisa e Inovação do Estado de Santa Catarina (FAPESC).

## Conflicts of Interest

The authors declare no conflicts of interest.

## Supporting information




**Supplementary Material—Figure S2** Sociodemographic description and profile of MS patients and the control group.

**Supplementary Material—Figure S4** Complete pairwise correlation analysis among clinical and biochemical variables in untreated patients with multiple sclerosis. All possible pairwise correlations among numerical variables were calculated. Spearman's rho, raw p‐values, Benjamini–Hochberg FDR‐adjusted p‐values, Pearson's *r*, and Pearson *p*‐values are shown. Spearman's correlation was considered the primary analysis, whereas Pearson's correlation was included as a sensitivity analysis.

**Supplementary Material—Figure S3** Complete Spearman correlation heatmap of clinical and biochemical variables in untreated patients with multiple sclerosis. The heatmap displays Spearman's correlation coefficients for all pairwise associations among clinical, inflammatory, oxidative stress, neurotrophic, neural injury, mitochondrial, mitophagy‐related, and apoptotic variables. Red indicates positive correlations and blue indicates negative correlations. Color intensity reflects the magnitude of Spearman's rho.

**Supplementary Material—Figure S5** Original, uncropped membrane images corresponding to the Western blot panels. The full membranes are provided to allow evaluation of band specificity, background signal, molecular weight position, and loading control quality. GAPDH was used as the loading control.

**Supplementary Material—Figure S1** List of primers used for Parkin, Pink, TFAM, and NRF‐1 analyses.

## Data Availability

Data will be made available upon plausible request to the corresponding author.

## References

[brb371707-bib-0002] Azoulay, D. , N. Urshansky , and A. Karni . 2008. “Low and Dysregulated BDNF Secretion From Immune Cells of MS Patients Is Related to Reduced Neuroprotection.” Journal of Neuroimmunology 195, no. 1–2: 186–193. 10.1016/j.jneuroim.2008.01.010. 18329726

[brb371707-bib-0003] Baldassarro, V. A. , A. Stanzani , L. Giardino , L. Calzà , and L. Lorenzini . 2022. “Neuroprotection and Neuroregeneration: Roles for the White Matter.” Neural Regeneration Research 17, no. 11: 2376–2380. 10.4103/1673-5374.335834. 35535874 PMC9120696

[brb371707-bib-0004] Beck, A. T. , R. A. Steer , and M. G. Carbin . 1988. “Psychometric Properties of the Beck Depression Inventory: Twenty‐five Years of Evaluation.” Clinical Psychology 8, no. 1: 77–100.

[brb371707-bib-0102] Beck, A. T. , and R. A. Steer . 1990. Manual for the Beck Anxiety Inventory. San Antonio, TX: The Psychological Corporation.

[brb371707-bib-0005] Bhat, A. H. , K. B. Dar , S. Anees , et al. 2015. “Oxidative Stress, Mitochondrial Dysfunction and Neurodegenerative Diseases; A Mechanistic Insight.” Biomedicine & Pharmacotherapy 74: 101–110. 10.1016/j.biopha.2015.07.025. 26349970

[brb371707-bib-0006] Bigaut, K. , J. De Seze , and N. Collongues . 2019. “Ocrelizumab for the Treatment of Multiple Sclerosis.” Expert Review of Neurotherapeutics 19, no. 2: 97–108. 10.1080/14737175.2019.1561284. 30570368

[brb371707-bib-0007] Boeschoten, R. E. , A. M. J. Braamse , A. T. F. Beekman , et al. 2017. “Prevalence of Depression and Anxiety in Multiple Sclerosis: A Systematic Review and Meta‐Analysis.” Journal of the Neurological Sciences 372: 331–341. 10.1016/j.jns.2016.11.067. 28017241

[brb371707-bib-0008] Bøyum, A. 1976. “Isolation of Lymphocytes, Granulocytes and Macrophages.” Scandinavian Journal of Immunology 5. no. Suppl 5: 9–15. 1052391

[brb371707-bib-0009] Campbell, G. R. , N. Ohno , D. M. Turnbull , and D. J. Mahad . 2012. “Mitochondrial Changes Within Axons in Multiple Sclerosis.” Current Opinion in Neurology 25, no. 3: 221–230. 10.1097/WCO.0b013e3283533a25. 22543429

[brb371707-bib-0011] Castellazzi, M. , S. Patergnani , M. Donadio , et al. 2019. “Correlation Between Auto/Mitophagic Processes and Magnetic Resonance Imaging Activity in Multiple Sclerosis Patients.” Journal of Neuroinflammation 16, no. 1: 131. 10.1186/s12974-019-1526-0. 31248423 PMC6598368

[brb371707-bib-0012] Chalah, M. A. , and S. S. Ayache . 2017. “Psychiatric Event in Multiple Sclerosis: Could It be the Tip of the Iceberg?” Revista Brasileira de Psiquiatria 39, no. 4: 365–368. 10.1590/1516-4446-2016-2105. 28355344 PMC7111399

[brb371707-bib-0014] Chitnis, T. , and H. L. Weiner . 2017. “CNS Inflammation and Neurodegeneration.” Journal of Clinical Investigation 127, no. 10: 3577–3587. 10.1172/jci90609. 28872464 PMC5617655

[brb371707-bib-0015] Cossu, D. , K. Yokoyama , L. A. Sechi , and N. Hattori . 2021. “Potential of PINK1 and PARKIN Proteins as Biomarkers for Active Multiple Sclerosis: A Japanese Cohort Study.” Frontiers in Immunology 12: 681386. 10.3389/fimmu.2021.681386. 34421896 PMC8371632

[brb371707-bib-0016] Dalgas, U. 2017. “Exercise Therapy in Multiple Sclerosis and Its Effects on Function and the Brain.” Neurodegenerative Disease Management 7, no. 6s: 35–40. 10.2217/nmt-2017-0040. 29143590

[brb371707-bib-0017] Dantzer, R. , J. C. O'Connor , G. G. Freund , R. W. Johnson , and K. W. Kelley . 2008. “From Inflammation to Sickness and Depression: When the Immune System Subjugates the Brain.” Nature Reviews Neuroscience 9, no. 1: 46–56. 10.1038/nrn2297. 18073775 PMC2919277

[brb371707-bib-0018] Dargahi, N. , M. Katsara , T. Tselios , et al. 2017. “Multiple Sclerosis: Immunopathology and Treatment Update.” Brain Sciences 7, no. 7: 78. 10.3390/brainsci7070078. 28686222 PMC5532591

[brb371707-bib-0019] De Angelis, F. , D. Plantone , and J. Chataway . 2018. “Pharmacotherapy in Secondary Progressive Multiple Sclerosis: An Overview.” CNS Drugs 32, no. 6: 499–526. 10.1007/s40263-018-0538-0. 29968175

[brb371707-bib-0020] De Meo, E. , E. Portaccio , and M. P. Amato . 2026. “Cognition in Multiple Sclerosis.” Current Opinion in Neurology 39, no. 3: 247–252. 10.1097/WCO.0000000000001481. 41960777 PMC13152055

[brb371707-bib-0021] Dias‐Carvalho, A. , M. Ferreira , A. Reis‐Mendes , et al. 2022. “Chemobrain: Mitoxantrone‐Induced Oxidative Stress, Apoptotic and Autophagic Neuronal Death in Adult CD‐1 Mice.” Archives of Toxicology 96, no. 6: 1767–1782. 10.1007/s00204-022-03261-x. 35306571

[brb371707-bib-0022] Dichev, V. , M. Kazakova , and V. Sarafian . 2020. “YKL‐40 and Neuron‐Specific Enolase in Neurodegeneration and Neuroinflammation.” Reviews in the Neurosciences 31, no. 5: 539–553. 10.1515/revneuro-2019-0100. 32045356

[brb371707-bib-0023] Di Sante, G. , S. Amadio , B. Sampaolese , et al. 2020. “The S100B Inhibitor Pentamidine Ameliorates Clinical Score and Neuropathology of Relapsing‐Remitting Multiple Sclerosis Mouse Model.” Cells 9, no. 3: 748. 10.3390/cells9030748. 32197530 PMC7140642

[brb371707-bib-0024] Donato, R. , B. R Cannon , G. Sorci , et al. 2013. “Functions of S100 Proteins.” Current Molecular Medicine 13, no. 1: 24–57. 10.2174/156652413804486214. 22834835 PMC3707951

[brb371707-bib-0025] Donato, R. , G. Sorci , F. Riuzzi , et al. 2009. “S100B's Double Life: Intracellular Regulator and Extracellular Signal.” Biochimica et Biophysica Acta (BBA)—Molecular Cell Research 1793, no. 6: 1008–1022. 10.1016/j.bbamcr.2008.11.009. 19110011

[brb371707-bib-0103] Dobryakova, E. , H. M. Genova , J. DeLuca , and G. R. Wylie . 2015. “The Dopamine Imbalance Hypothesis of Fatigue in Multiple Sclerosis and Other Neurological Disorders.” Frontiers in Neurology 6: 52. 25814977 10.3389/fneur.2015.00052 PMC4357260

[brb371707-bib-0026] Draper, H. H. , and M. Hadley . 1990. “Malondialdehyde Determination as Index of Lipid Peroxidation.” Methods in Enzymology 186: 421–431. 10.1016/0076-6879(90)86135-i. 2233309

[brb371707-bib-0027] Eftekhari, E. , M. Mostahfezian , M. Etemadifar , and A. Zafari . 2012. “Resistance Training and Vibration Improve Muscle Strength and Functional Capacity in Female Patients With Multiple Sclerosis.” Asian Journal of Sports Medicine 3, no. 4: 279–284. 10.5812/asjsm.34552. 23342227 PMC3525825

[brb371707-bib-0028] Elliott, E. I. , and F. S. Sutterwala . 2015. “Initiation and Perpetuation of NLRP 3 Inflammasome Activation and Assembly.” Immunological Reviews 265, no. 1: 35–52. 10.1111/imr.12286. 25879282 PMC4400874

[brb371707-bib-0029] Fischer, J. S. , N. G. LaRocca , D. M. Miller , P. G. Ritvo , H. Andrews , and D. Paty . 1999. “Recent Developments in the Assessment of Quality of Life in Multiple Sclerosis (MS).” Multiple Sclerosis Journal 5, no. 4: 251–259. 10.1177/135245859900500410. 10467384

[brb371707-bib-0030] Forooghian, F. , R. K. Cheung , W. C. Smith , P. O'connor , and H.‐M. Dosch . 2007. “Enolase and Arrestin Are Novel Nonmyelin Autoantigens in Multiple Sclerosis.” Journal of Clinical Immunology 27, no. 4: 388–396. 10.1007/s10875-007-9091-1. 17436063 PMC2705966

[brb371707-bib-0031] Frota, E. R. , D. H. Rodrigues , E. A. Donadi , D. G. Brum , D. R. Maciel , and A. L. Teixeira . 2009. “Increased Plasma Levels of Brain Derived Neurotrophic Factor (BDNF) After Multiple Sclerosis Relapse.” Neuroscience Letters 460, no. 2: 130–132. 10.1016/j.neulet.2009.05.057. 19477225

[brb371707-bib-0032] Furlan, R. , G. Martino , F. Galbiati , et al. 1999. “Caspase‐1 Regulates the Inflammatory Process Leading to Autoimmune Demyelination.” The Journal of Immunology 163, no. 5: 2403–2409. 10.4049/jimmunol.163.5.2403. 10452974

[brb371707-bib-0033] García‐Ruiz, I. , P. Solís‐Muñoz , D. Fernández‐Moreira , T. Muñoz‐Yagüe , and J. A. Solís‐Herruzo . 2013. “Pioglitazone Leads to an Inactivation and Disassembly of Complex I of the Mitochondrial Respiratory Chain.” BMC Biology 11: 88. 10.1186/1741-7007-11-88. 23915000 PMC3751493

[brb371707-bib-0034] Gavrilă, M. G. , C. V. Albu , B. C. Albu , E. Burada , R. E. Sandu , and R. Surugiu . 2026. “The Crossroads of Neuroinflammation and Biomarkers in Multiple Sclerosis: A Systematic Review.” Cells 15, no. 7: 610. 10.3390/cells15070610. 41972700 PMC13072207

[brb371707-bib-0035] Giordano, C. , L. Iommarini , L. Giordano , et al. 2014. “Efficient Mitochondrial Biogenesis Drives Incomplete Penetrance in Leber's Hereditary Optic Neuropathy.” Brain 137, no. Pt 2: 335–353. 10.1093/brain/awt343. 24369379 PMC3914475

[brb371707-bib-0036] Gold, S. M. , S. Kruger , K. J. Ziegler , et al. 2011. “Endocrine and Immune Substrates of Depressive Symptoms and Fatigue in Multiple Sclerosis Patients With Comorbid Major Depression.” Journal of Neurology, Neurosurgery & Psychiatry 82, no. 7: 814–818. 10.1136/jnnp.2010.230029. 21296901

[brb371707-bib-0037] Gómez‐Melero, S. , J. Caballero‐Villarraso , B. M. Escribano , A. Galvao‐Carmona , I. Túnez , and E. Agüera‐Morales . 2024. “Impact of Cognitive Impairment on Quality of Life in Multiple Sclerosis Patients—A Comprehensive Review.” Journal of Clinical Medicine 13, no. 11: 3321. 10.3390/jcm13113321. 38893032 PMC11172944

[brb371707-bib-0038] Gottschalk, M. , T. Kümpfel , P. Flachenecker , et al. 2005. “Fatigue and Regulation of the Hypothalamo‐pituitary‐adrenal Axis in Multiple Sclerosis.” Archives of Neurology 62, no. 2: 277. 10.1001/archneur.62.2.277. 15710856

[brb371707-bib-0039] Gris, D. , Z. Ye , H. A. Iocca , et al. 2010. “NLRP3 Plays a Critical Role in the Development of Experimental Autoimmune Encephalomyelitis by Mediating Th1 and Th17 Responses.” The Journal of Immunology 185, no. 2: 974–981. 10.4049/jimmunol.0904145. 20574004 PMC3593010

[brb371707-bib-0040] Håkansson, I. , A. Tisell , P. Cassel , et al. 2017. “Neurofilament Light Chain in Cerebrospinal Fluid and Prediction of Disease Activity in Clinically Isolated Syndrome and Relapsing–Eemitting Multiple Sclerosis.” European Journal of Neurology 24, no. 5: 703–712. 10.1111/ene.13274. 28261960

[brb371707-bib-0041] Harding, A. E. , M. G. Sweeney , D. H. Miller , et al. 1992. “Occurrence of a Multiple Sclerosis‐Like Illness in Women Who Have a Leber'S Hereditary Optic Neuropathy Mitochondrial DNA Mutation.” Brain 115, no. Pt 4: 979–989. 10.1093/brain/115.4.979. 1393514

[brb371707-bib-0042] Hayashi, G. , M. Jasoliya , S. Sahdeo , et al. 2017. “Dimethyl Fumarate Mediates Nrf2‐dependent Mitochondrial Biogenesis in Mice and Humans.” Human Molecular Genetics 26, no. 15: 2864–2873. 10.1093/hmg/ddx167. 28460056 PMC6251607

[brb371707-bib-0043] Heesen, C. , S. M. Gold , S. Hartmann , et al. 2003. “Endocrine and Cytokine Responses to Standardized Physical Stress in Multiple Sclerosis.” Brain, Behavior, and Immunity 17, no. 6: 473–481. 10.1016/S0889-1591(03)00077-1. 14583239

[brb371707-bib-0104] Heitmann, H. , T. F. Andlauer , T. Korn , et al. 2020. “Fatigue, Depression, and Pain in Multiple Sclerosis: How Neuroinflammation Translates into Dysfunctional Reward Processing and Anhedonic Symptoms.” Multiple Sclerosis Journal 26, no. 13: 1647–1658. 33179588 10.1177/1352458520972279 PMC9131410

[brb371707-bib-0044] Hemmerich, M. , N. Malorny , A. Lewen , J. O. Hollnagel , B. Chausse , and O. Kann . 2022. “Priming of Microglia by Type II Interferon Is Lasting and Resistant to Modulation by Interleukin‐10 in Situ.” Journal of Neuroimmunology 368: 577881. 10.1016/j.jneuroim.2022.577881. 35537331

[brb371707-bib-0045] Hokari, M. , A. Yokoseki , M. Arakawa , et al. 2016. “Clinicopathological Features in Anterior Visual Pathway in Neuromyelitis Optica.” Annals of Neurology 79, no. 4: 605–624. 10.1002/ana.24608. 26836302

[brb371707-bib-0046] Horng, T. J. 2014. “Calcium Signaling and Mitochondrial Destabilization in the Triggering of the NLRP3 Inflammasome.” Trends in Immunology 35, no. 6: 253–261. 10.1016/j.it.2014.02.007. 24646829 PMC4041823

[brb371707-bib-0047] Howard, J. F. , R. J. Nowak , G. I. Wolfe , et al. 2020. “Clinical Effects of the Self‐administered Subcutaneous Complement Inhibitor Zilucoplan in Patients With Moderate to Severe Generalized Myasthenia Gravis: Results of a Phase 2 Randomized, Double‐Blind, Placebo‐Controlled, Multicenter Clinical Trial.” JAMA Neurology 77, no. 5: 582. 10.1001/jamaneurol.2019.5125. 32065623 PMC7042797

[brb371707-bib-0048] Huang, Y. , and C. F. Dreyfus . 2016. “The Role of Growth Factors as a Therapeutic Approach to Demyelinating Disease.” Experimental Neurology 283, no. Pt B: 531–540. 10.1016/j.expneurol.2016.02.023. 27016070 PMC5010931

[brb371707-bib-0049] Inoue, M. , and M. L. Shinohara . 2013. “NLRP3 Inflammasome and MS/EAE.” Autoimmune Diseases 2013: 859145. 10.1155/2013/859145. 23365725 PMC3556409

[brb371707-bib-0050] Isgrò, M. A. , P. Bottoni , and R. Scatena . 2015. “Neuron‐Specific Enolase as a Biomarker: Biochemical and Clinical Aspects.” Advances in Experimental Medicine and Biology 867: 125–143. 10.1007/978-94-017-7215-0_9. 26530364

[brb371707-bib-0051] Jiménez‐Zambrano, J. , K. Zambrano‐Llaguno , and M. G. Acuña‐Chong . 2020. “Social Anxiety Disorder Among Patients With Multiple Sclerosis: A Population‐Based Case‐Control Study in Ecuador.” Revista De Neurologia 70, no. 2: 45–52. 10.33588/rn.7002.2019213. 31930470

[brb371707-bib-0052] Julian, L. J. , and P. A. Arnett . 2009. “Relationships Among Anxiety, Depression, and Executive Functioning in Multiple Sclerosis.” The Clinical Neuropsychologist 23, no. 5: 794–804. 10.1080/13854040802665808. 19241295

[brb371707-bib-0053] Khademosharie, M. , V. Tadibi , N. Behpoor , and M. R. Hamedinia . 2018. “The Effect of 12‐weeks Concurent Training on the Serum Levels NGF, BDNF, and VDBP in Women With Multiple Sclerosis.” International Journal of Applied Exercise Physiology 7, no. 1: 77–86. 10.22631/ijaep.v7i1.228.

[brb371707-bib-0054] Ko, W. H. , J. Hyneck , and J. Homa . 1979. “Single Frequency RF Powered ECG Telemetry System.” IEEE Transactions on Biomedical Engineering 26, no. 2: 105–109. 10.1109/tbme.1979.326518. 104921

[brb371707-bib-0055] Koch, M. W. , S. George , W. Wall , V. Wee Yong , and L. M. Metz . 2015. “Serum NSE Level and Disability Progression in Multiple Sclerosis.” Journal of the Neurological Sciences 350, no. 1–2: 46–50. 10.1016/j.jns.2015.02.009. 25686504

[brb371707-bib-0056] Kothari, P. H. , G. R. Kolar , J. C. Jen , et al. 2018. “TREX 1 Is Expressed by Microglia in Normal Human Brain and Increases in Regions Affected by Ischemia.” Brain Pathology 28, no. 6: 806–821. 10.1111/bpa.12626. 30062819 PMC6404532

[brb371707-bib-0057] Krupp, L. B. , N. G. LaRocca , J. Muir‐Nash , and A. D. Steinberg . 1989. “The Fatigue Severity Scale. Application to Patients With Multiple Sclerosis and Systemic Lupus Erythematosus.” Archives of Neurology 46, no. 10: 1121. 10.1001/archneur.1989.00520460115022. 2803071

[brb371707-bib-0058] Kurihara, T. , M. Narita , H. Kawaradani , T. Kikkawa , and M. Usami . 1979. “[Radionuclide Assessment of Nitroglycerin Influence on Regional Myocardial Perfusion in Ischemic Heart (author's transl)].” Kokyu to Junkan Respiration & Circulation 27, no. 9: 999–1006. 119987

[brb371707-bib-0059] Le, H. H. , J. Ken‐Opurum , A. LaPrade , M. C. Maculaitis , and J. J. Sheehan . 2024. “Exploring Humanistic Burden of Fatigue in Adults With Multiple Sclerosis: An Analysis of US National Health and Wellness Survey Data.” BMC Neurology 24, no. 1: 51. 10.1186/s12883-023-03423-z. 38297247 PMC10832085

[brb371707-bib-0060] Levine, R. L. , D. Garland , C. N. Oliver , et al. 1990. “Determination of Carbonyl Content in Oxidatively Modified Proteins.” Methods in Enzymology 186: 464–478. 10.1016/0076-6879(90)86141-H. 1978225

[brb371707-bib-0061] Lowry, O. H. , N. J. Rosebrough , A. L. Farr , and R. J. Randall . 1951. “Protein Measurement With the Folin Phenol Reagent.” Journal of Biological Chemistry 193, no. 1: 265–275. 10.1016/S0021-9258(19)52451-6. 14907713

[brb371707-bib-0063] Mahad, D. , H. Lassmann , and D. Turnbull . 2008. “Review: Mitochondria and Disease Progression in Multiple Sclerosis.” Neuropathology and Applied Neurobiology 34, no. 6: 577–589. 10.1111/j.1365-2990.2008.00987.x. 19076696 PMC2981078

[brb371707-bib-0064] Malhotra, S. , C. Costa, H. Eixarch, et al. 2020. “NLRP3 inflammasome as a Pathogenic Driver and Therapeutic Target in Multiple Sclerosis and Experimental Autoimmune Encephalomyelitis.” Frontiers in Immunology 11: 1167. 10.3389/fimmu.2020.01167. 32595638 PMC7300210

[brb371707-bib-0065] Mao, P. , and P. H. Reddy . 2010. “Is Multiple Sclerosis a Mitochondrial Disease?” Biochimica et Biophysica Acta (BBA)—Molecular Basis of Disease 1802, no. 1: 66–79. 10.1016/j.bbadis.2009.07.002. 19607913 PMC2790545

[brb371707-bib-0066] Mendes, B. , T. Firmino , R. Oliveira , et al. 2020. “Effects of Knee Flexor Submaximal Isometric Contraction Until Exhaustion on Semitendinosus and Biceps Femoris Long Head Shear Modulus in Healthy Individuals.” Scientific Reports 10, no. 1: 16433. 10.1038/s41598-020-73433-1. 33009453 PMC7532170

[brb371707-bib-0067] Michels, M. , M. R. Abatti , P. Ávila , et al. 2020. “Characterization and Modulation of Microglial Phenotypes in an Animal Model of Severe Sepsis.” Journal of Cellular and Molecular Medicine 24, no. 1: 88–97. 10.1111/jcmm.14606. 31654493 PMC6933367

[brb371707-bib-0068] Miller, A. H. , E. Haroon , C. L. Raison , and J. C. Felger . 2013. “Cytokine Targets in the Brain: Impact on Neurotransmitters and Neurocircuits.” Depression and Anxiety 30, no. 4: 297–306. 10.1002/da.22084. 23468190 PMC4141874

[brb371707-bib-0069] Mills, R. , C. Young , R. Nicholas , J. Pallant , and A. Tennant . 2009. “Rasch Analysis of the Fatigue Severity Scale in Multiple Sclerosis.” Multiple Sclerosis Journal 15, no. 1: 81–87. 10.1177/1352458508096215. 18755824

[brb371707-bib-0070] Morris, G. , B. K. Puri , K. Walder , et al. 2018. “The Endoplasmic Reticulum Stress Response in Neuroprogressive Diseases: Emerging Pathophysiological Role and Translational Implications.” Molecular Neurobiology 55, no. 12: 8765–8787. 10.1007/s12035-018-1028-6. PMC6208857 29594942

[brb371707-bib-0071] Morrow, S. A. 2018. “Anxiety Is More Important Than Depression in MS—Yes.” Multiple Sclerosis Journal 24, no. 4: 440–441. 10.1177/1352458517751652. 29381115

[brb371707-bib-0072] Murdaca, G. , A. Tonacci , S. Negrini , et al. 2019. “Emerging Role of Vitamin D in Autoimmune Diseases: An Update on Evidence and Therapeutic Implications.” Autoimmunity Reviews 18, no. 9: 102350. 10.1016/j.autrev.2019.102350. 31323357

[brb371707-bib-0073] Naegelin, Y. , K. Saeuberli , S. Schaedelin , et al. 2020. “Levels of Brain‐Derived Neurotrophic Factor in Patients With Multiple Sclerosis.” Annals of Clinical and Translational Neurology 7, no. 11: 2251–2261. 10.1002/acn3.51215. 33031634 PMC7664260

[brb371707-bib-0074] Nociti, V. 2020. “What Is the Role of Brain Derived Neurotrophic Factor in Multiple Sclerosis Neuroinflammation?” Neuroimmunology and Neuroinflammation 7, no. 3: 291–299.

[brb371707-bib-0075] Ofengeim, D. , Y. Ito , A. Najafov , et al. 2015. “Activation of Necroptosis in Multiple Sclerosis.” Cell Reports 10, no. 11: 1836–1849. 10.1016/j.celrep.2015.02.051. 25801023 PMC4494996

[brb371707-bib-0076] Olcum, M. , B. Tastan , C. Kiser , S. Genc , and K. Genc . 2020. “Microglial NLRP3 Inflammasome Activation in Multiple Sclerosis.” Advances in Protein Chemistry and Biology 119: 247–308. 10.1016/bs.apcsb.2019.08.007. 31997770

[brb371707-bib-0077] Oliva Ramirez, A. , A. Keenan , O. Kalau , E. Worthington , L. Cohen , and S. Singh . 2021. “Prevalence and Burden of Multiple Sclerosis‐Related Fatigue: A Systematic Literature Review.” BMC Neurology 21, no. 1: 468. 10.1186/s12883-021-02396-1. 34856949 PMC8638268

[brb371707-bib-0078] Paknejad, B. , H. Shirkhanloo , and M. Aliomrani . 2019. “Is There any Relevance Between Serum Heavy Metal Concentration and BBB Leakage in Multiple Sclerosis Patients?” Biological Trace Element Research 190, no. 2: 289–294. 10.1007/s12011-018-1553-1. 30368653

[brb371707-bib-0079] Panda, S. P. , R. C. Das , K. Srivastava , A. Ratnam , and N. Sharma . 2018. “Psychiatric Comorbidity in Multiple Sclerosis.” Neurologia i Neurochirurgia Polska 52, no. 6: 704–709. 10.1016/j.pjnns.2018.09.003. 30274945

[brb371707-bib-0080] Patergnani, S. , M. Castellazzi , M. Bonora , et al. 2018. “Autophagy and Mitophagy Elements Are Increased in Body Fluids of Multiple Sclerosis‐Affected Individuals.” Journal of Neurology, Neurosurgery & Psychiatry 89, no. 4: 439–441. 10.1136/jnnp-2017-316234. 28866627

[brb371707-bib-0081] Pavan, K. , K. Schmidt , B. Marangoni , M. F. Mendes , C. P. Tilbery , and S. Lianza . 2007. “Esclerose Múltipla: Adaptação Transcultural e Validação da Escala Modificada de Impacto de Fadiga.” Arquivos de Neuro‐Psiquiatria 65, no. 3a: 669–673. 10.1590/s0004-2822007000400024. 17876412

[brb371707-bib-0082] Petersen, E. N. , E. Bechgaard , R. J. Sortwell , and L. Wetterberg . 1978. “Potent Depletion of 5HT From Monkey Whole Bloob by a New 5HT Uptake Inhibitor, Paroxetine (FG 7051).” European Journal of Pharmacology 52, no. 1: 115–119. 10.1016/0014-2999(78)90028-6. 102515

[brb371707-bib-0083] Pfeffer, G. , A. Burke , P. Yu‐Wai‐Man , D. A. Compston , and P. F. Chinnery . 2013. “Clinical Features of MS Associated With Leber Hereditary Optic Neuropathy mtDNA Mutations.” Neurology 81, no. 24: 2073–2081. 10.1212/01.wnl.0000437308.22603.43. 24198293 PMC3863351

[brb371707-bib-0084] Reese, J. P. , G. Wienemann , A. John , et al. 2013. “Preference‐Based Health Status in a German Outpatient Cohort With Multiple Sclerosis.” Health and Quality of Life Outcomes 11: 162. 10.1186/1477-7525-11-162. 24089999 PMC3851447

[brb371707-bib-0085] Reich, D. S. , C. F. Lucchinetti , and P. A. Calabresi . 2018. “Multiple Sclerosis.” New England Journal of Medicine 378, no. 2: 169–180. 10.1056/NEJMra1401483. 29320652 PMC6942519

[brb371707-bib-0086] Restuccia, G. , C. Susinna , G. Marafioti , et al. 2025. “Serum and Cerebrospinal Fluid Biomarkers as Predictors of Cognitive Impairment in Multiple Sclerosis: A Systematic Review of Longitudinal Studies.” Journal of Neurology 273, no. 1: 36. 10.1007/s00415-025-13499-x. 41428098 PMC12722371

[brb371707-bib-0087] Rojas, J. I. , A. Pappolla , L. Patrucco , et al. 2023. “Disability Outcomes in NMOSD and MOGAD Patients: Data From a Nationwide Registry in Argentina.” Neurological Sciences 44, no. 1: 281–286. 10.1007/s10072-022-06409-w. 36166174

[brb371707-bib-0088] Rossi, S. , V. Studer , C. Motta , et al. 2017. “Neuroinflammation Drives Anxiety and Depression in Relapsing‐Remitting Multiple Sclerosis.” Neurology 88, no. 14: 1338–1347. 10.1212/WNL.0000000000004411. 28842450

[brb371707-bib-0089] Scheller, F. , M. Jänchen , J. Lampe , H. J. Prümke , J. Blanck , and E. Palecek . 1975. “Studies on Electron Transfer Between Mercury Electrode and Hemoprotein.” Biochimica et Biophysica Acta (BBA)—Protein Structure 412, no. 1: 157–167. 10.1016/0005-2795(75)90348-7. 79

[brb371707-bib-0090] Scheu, S. , S. Ali , R. Mann‐Nüttel , et al. 2019. “Interferon β‐Mediated Protective Functions of Microglia in Central Nervous System Autoimmunity.” International Journal of Molecular Sciences 20, no. 1: 190. 10.3390/ijms20010190. 30621022 PMC6337097

[brb371707-bib-0091] Shao, S. , C. Chen , G. Shi , et al. 2021. “Therapeutic Potential of the Target on NLRP3 Inflammasome in Multiple Sclerosis.” Pharmacology & Therapeutics 227: 107880. 10.1016/j.pharmthera.2021.107880. 33901504

[brb371707-bib-0092] Shenker, B. J. , D. M. Ojcius , L. P. Walker , A. Zekavat , M. D. Scuron , and K. Boesze‐Battaglia . 2015. “Aggregatibacter Actinomycetemcomitans Cytolethal Distending Toxin Activates the NLRP3 Inflammasome in Human Macrophages, Leading to the Release of Proinflammatory Cytokines.” Infection and immunity 83, no. 4: 1487–1496. 10.1128/IAI.03132-14. 25644004 PMC4363449

[brb371707-bib-0093] Shi, F.‐D. , K. Takeda , S. Akira , N. Sarvetnick , and H.‐G. Ljunggren . 2000. “IL‐18 Directs Autoreactive T Cells and Promotes Autodestruction in the Central Nervous System via Induction of IFN‐γ by NK Cells.” The Journal of Immunology 165, no. 6: 3099–3104. 10.4049/jimmunol.165.6.3099. 10975822

[brb371707-bib-0094] Shobeiri, P. , A. Karimi , S. Momtazmanesh , et al. 2022. “Exercise‐Induced Increase in Blood‐Based Brain‐Derived Neurotrophic Factor (BDNF) in People With Multiple Sclerosis: A Systematic Review and Meta‐Analysis of Exercise Intervention Trials.” PLoS ONE 17, no. 3: e0264557. 10.1371/journal.pone.0264557. 35239684 PMC8893651

[brb371707-bib-0095] Stadelmann, C. , S. Timmler , A. Barrantes‐Freer , and M. Simons . 2019. “Myelin in the Central Nervous System: Structure, Function, and Pathology.” Physiological Reviews 99, no. 3: 1381–1431. 10.1152/physrev.00031.2018. 31066630

[brb371707-bib-0097] Wessels, I. , and L. Rink . 2020. “Micronutrients in Autoimmune Diseases: Possible Therapeutic Benefits of Zinc and Vitamin D.” The Journal of Nutritional Biochemistry 77: 108240. 10.1016/j.jnutbio.2019.108240. 31841960

[brb371707-bib-0098] Yamout, B. I. , and R. Alroughani . 2018. “Multiple Sclerosis.” Seminars in Neurology 38, no. 2: 212–225. 10.1055/s-0038-1649502. 29791948

[brb371707-bib-0099] Yao, R. Q. , C. Ren , Z. F. Xia , and Y. M. Yao . 2021. “Organelle‐specific Autophagy in Inflammatory Diseases: a Potential Therapeutic Target Underlying the Quality Control of Multiple Organelles.” Autophagy 17, no. 2: 385–401. 10.1080/15548627.2020.1725377. 32048886 PMC8007140

[brb371707-bib-0100] Yi, X. , Y. Zhang , Q. Du , et al. 2024. “Global Prevalence of Fatigue in Patients With Multiple Sclerosis: A Systematic Review and Meta‐Analysis.” Frontiers in Neurology 15: 1457788. 10.3389/fneur.2024.1457788. 39416662 PMC11479926

[brb371707-bib-0101] Zveik, O. , N. Fainstein , A. Rechtman , et al. 2022. “Cerebrospinal Fluid of Progressive Multiple Sclerosis Patients Reduces Differentiation and Immune Functions of Oligodendrocyte Progenitor Cells.” Glia 70, no. 6: 1191–1209. 10.1002/glia.24165. 35266197 PMC9314832

