# Supplementary material for: Associations Between Neuromuscular Fatigue, Anxiety, and Peripheral Biomarkers of Neuronal Injury, Inflammation, and Mitochondrial Dysfunction in Treatment‐Naïve Relapsing‐Remitting Multiple Sclerosis
Source: Brain Behav. 2026 Aug 25;16(8):e71707. doi: 10.1002/brb3.71707 (PMC13507521; doi:10.1002/brb3.71707)
Supplement: Supplementary file 1 [file BRB3-16-e71707-s002.pdf]

Supplementary Material – Figure 5 - Original, uncropped membrane images corresponding to the Western blot panels

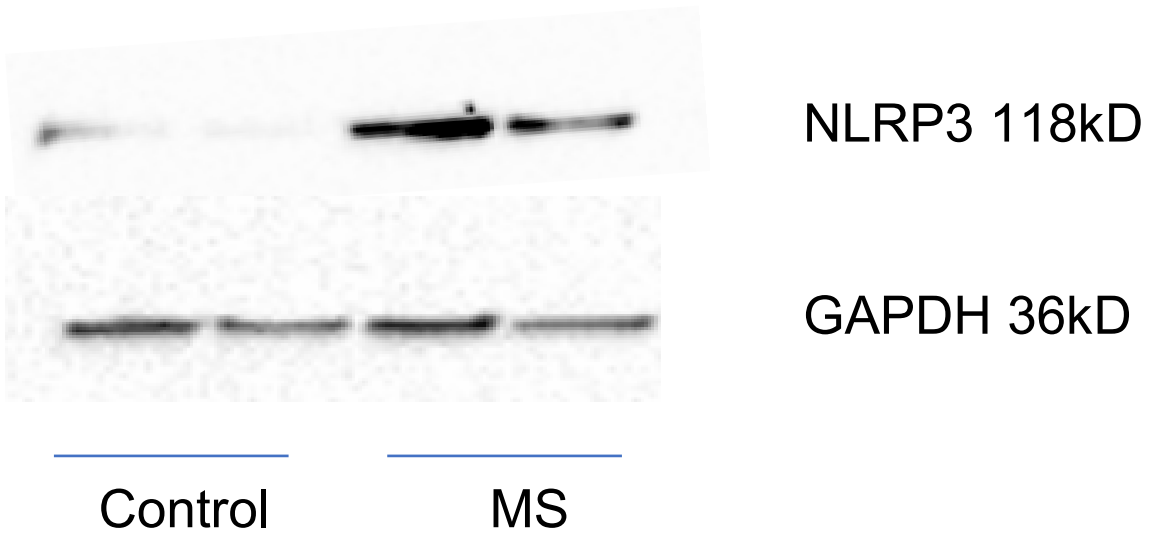

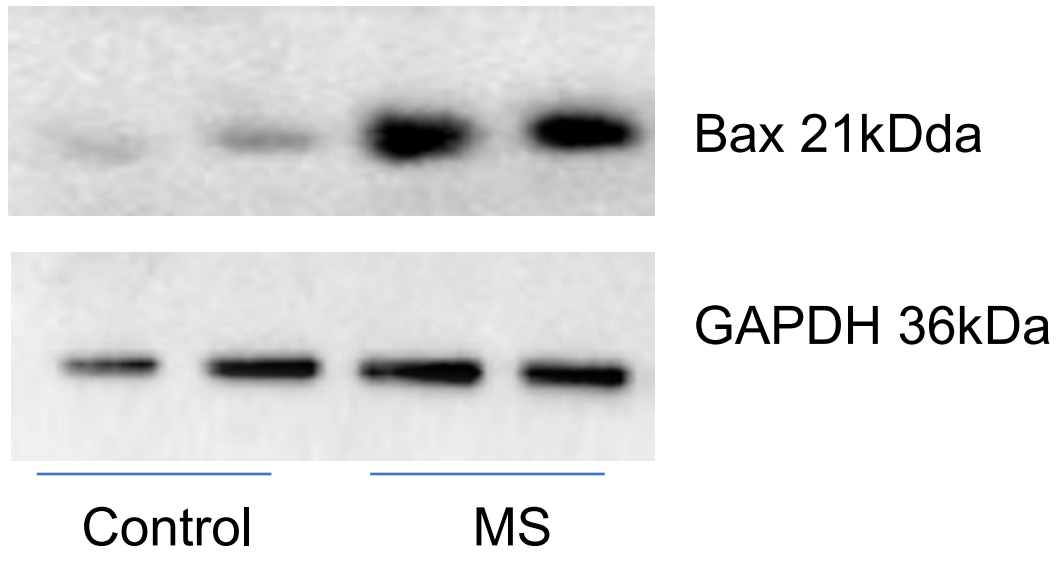

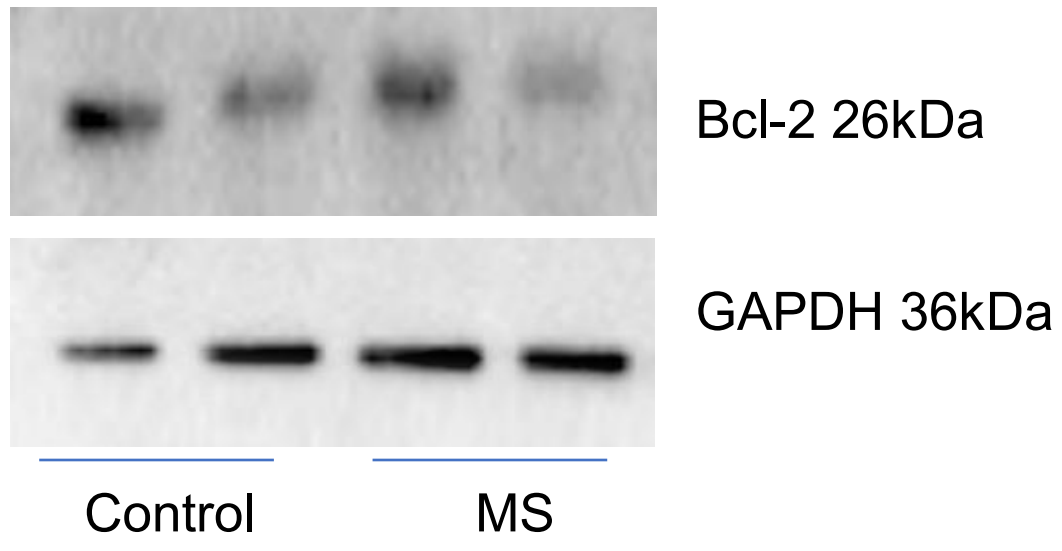

Bax

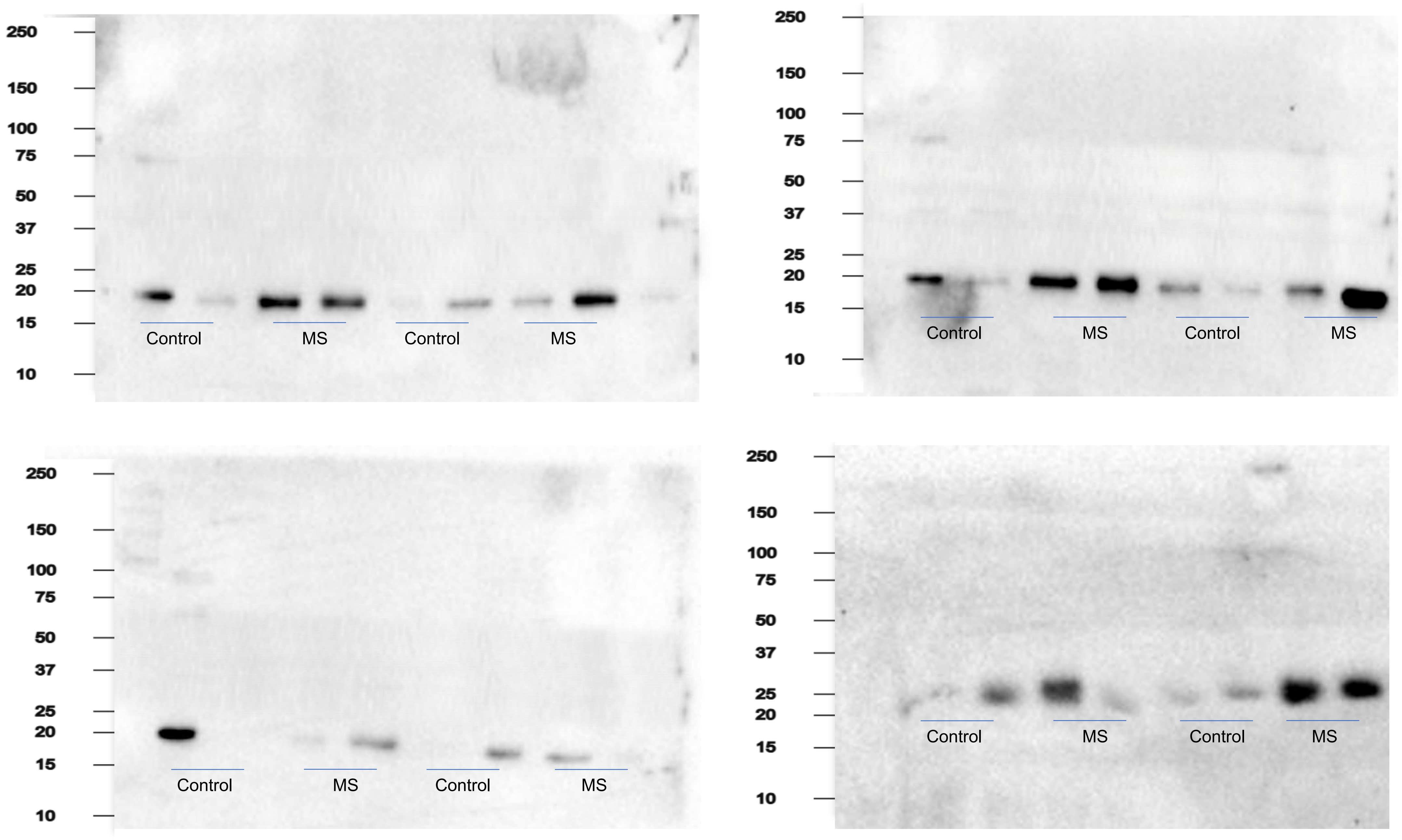
250
150
100
75
50
37
25
20
15
10
Control
MS
Control
MS
250
150
100
75
50
37
25
20
15
10
Control
MS
Control
MS
250
150
100
75
50
37
25
20
15
10
Control
MS
Control
MS
250
150
100
75
50
37
25
20
15
10
Control
MS
Control
MS

BCL-2

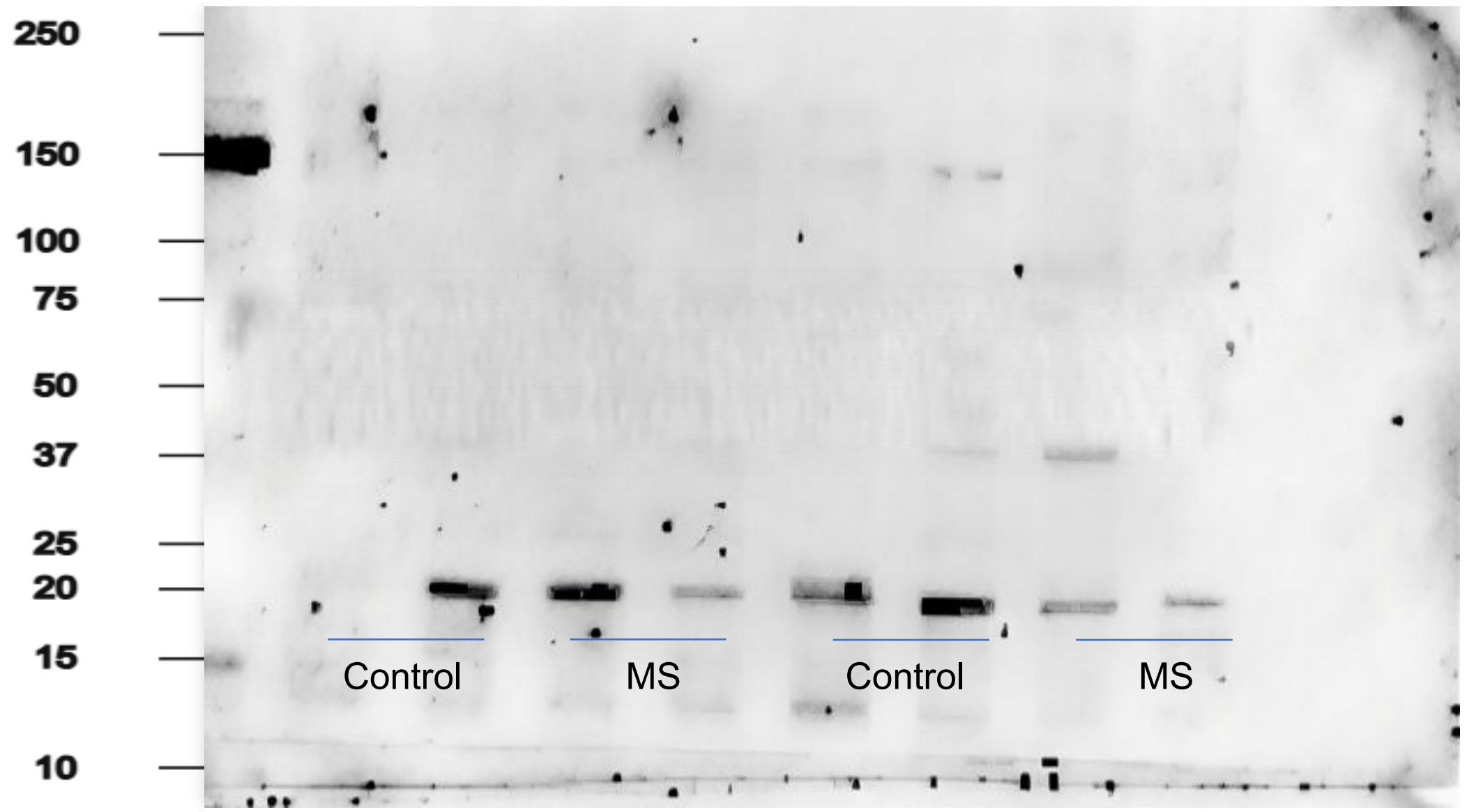
250
150
100
75
50
37
25
20
15
10
Control
MS
Control
MS

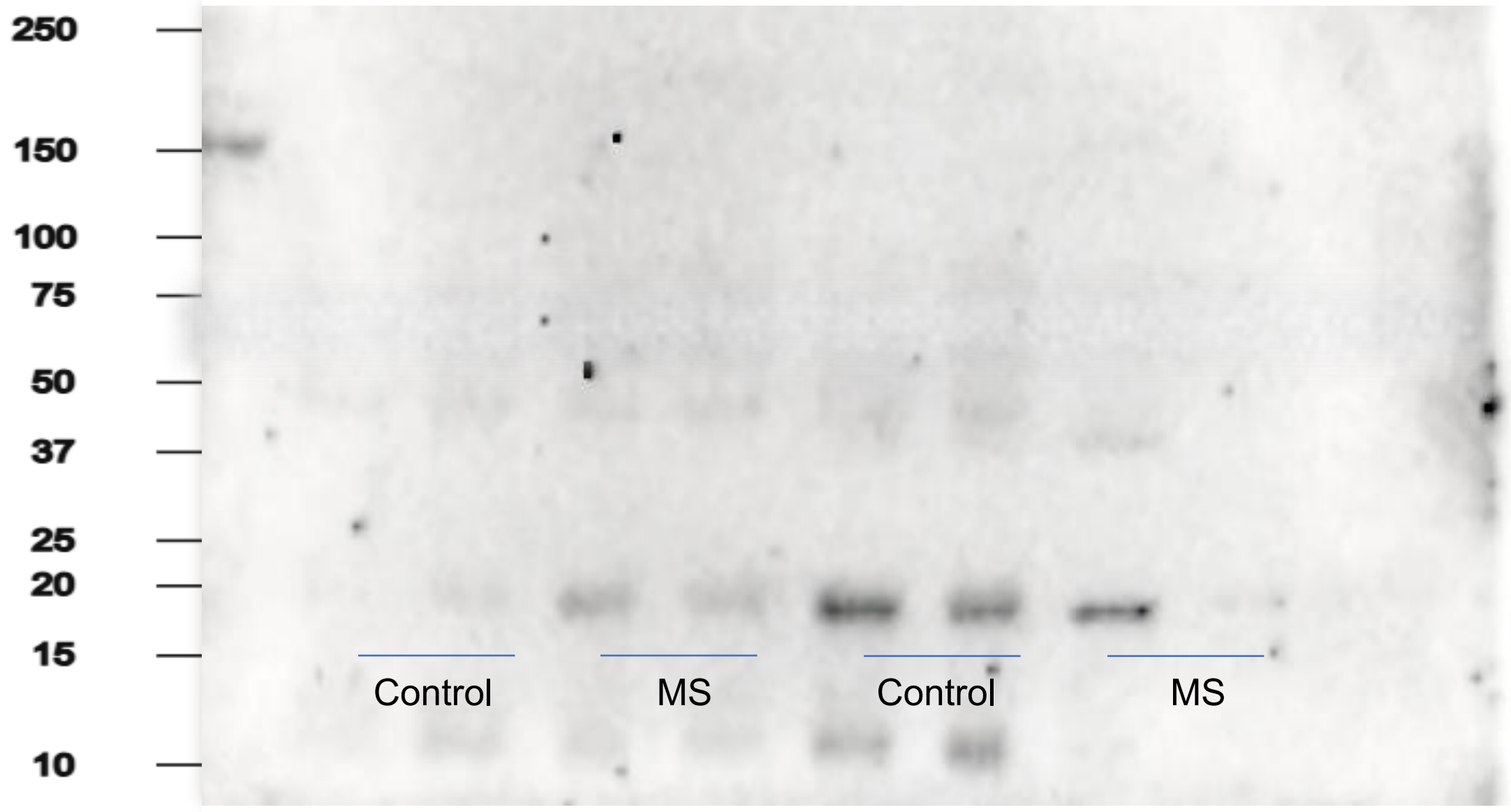
250
150
100
75
50
37
25
20
15
10
Control
MS
Control
MS

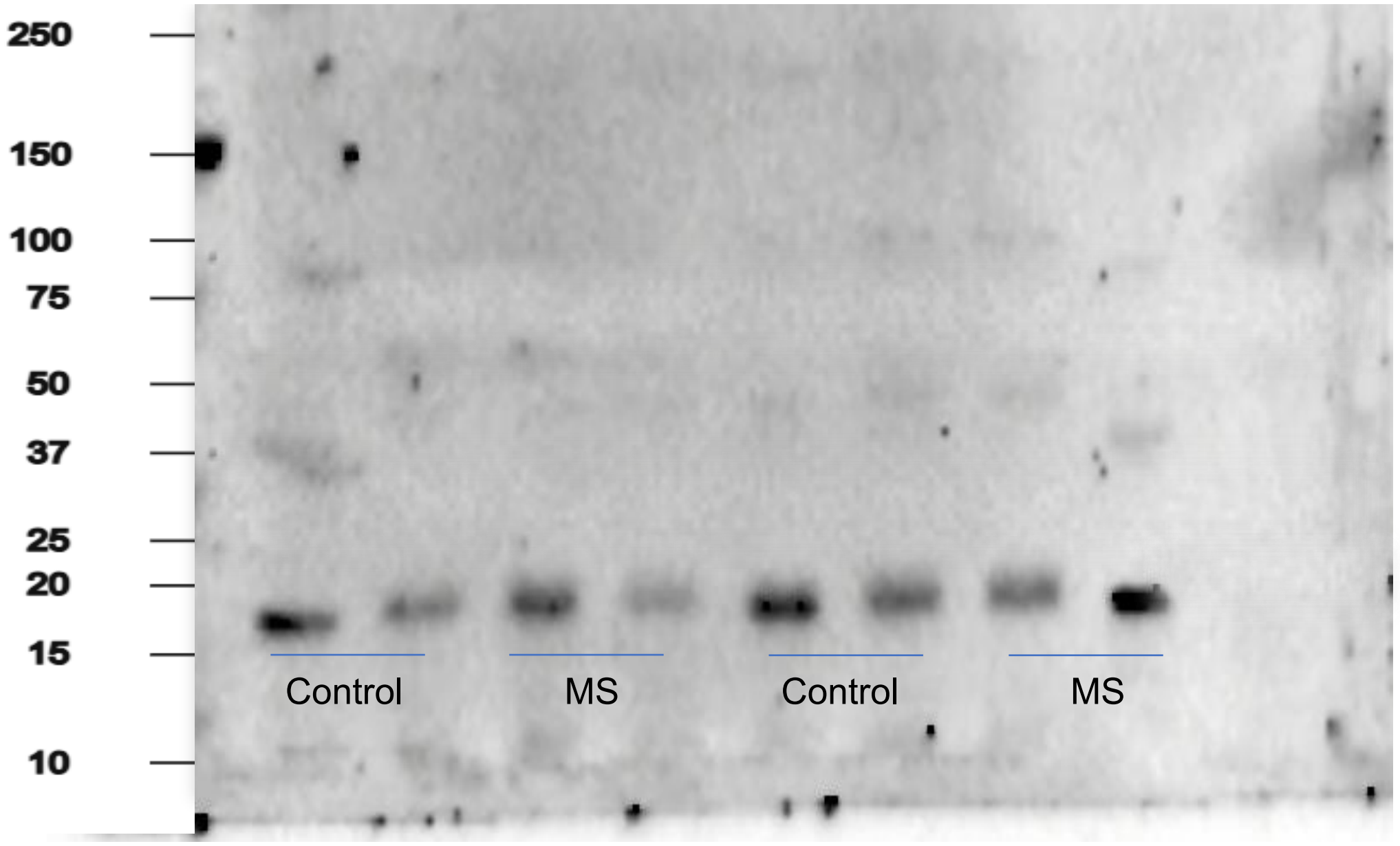
250
150
100
75
50
37
25
20
15
10
Control
MS
Control
MS

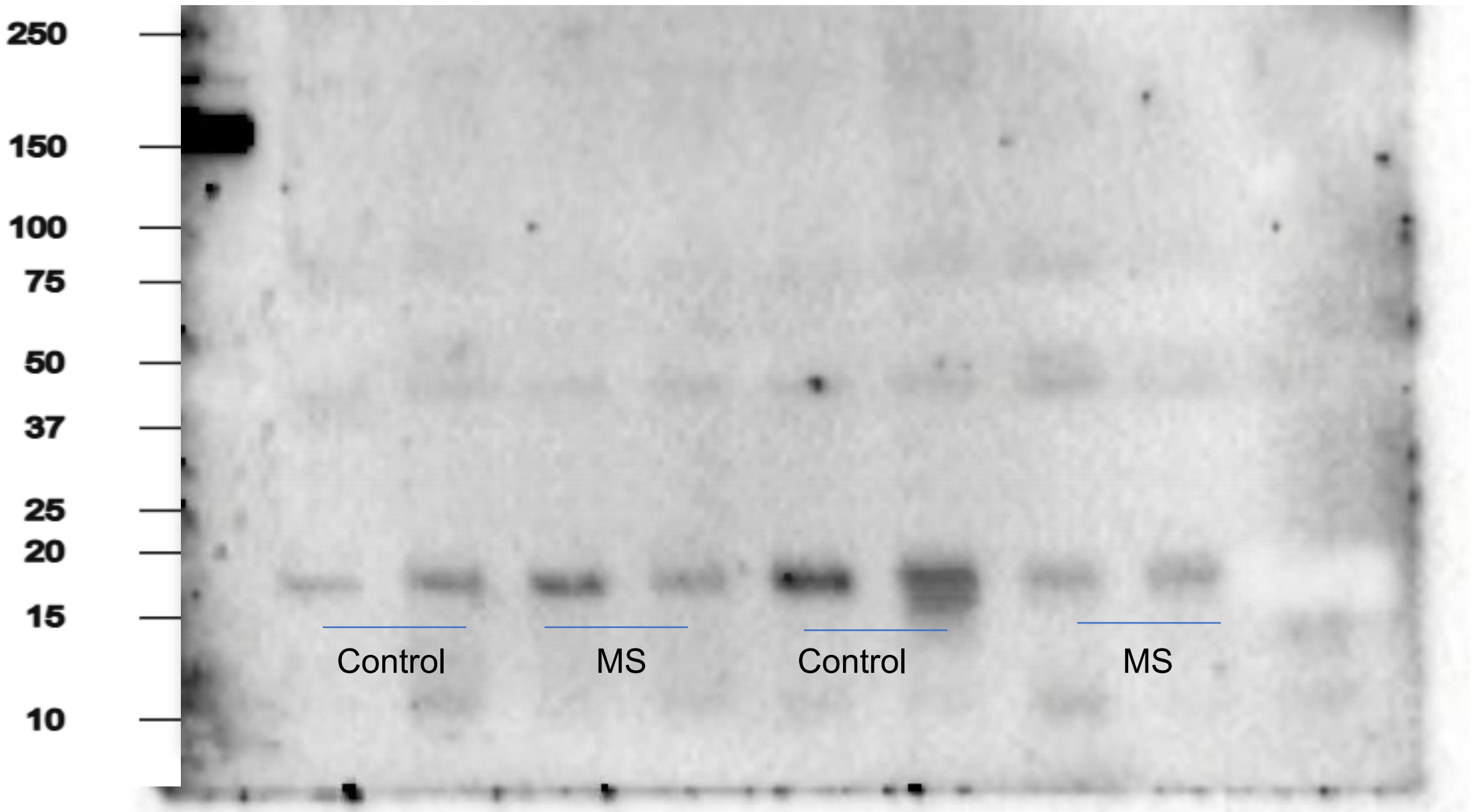
250
150
100
75
50
37
25
20
15
10
Control
MS
Control
MS

GAPDH

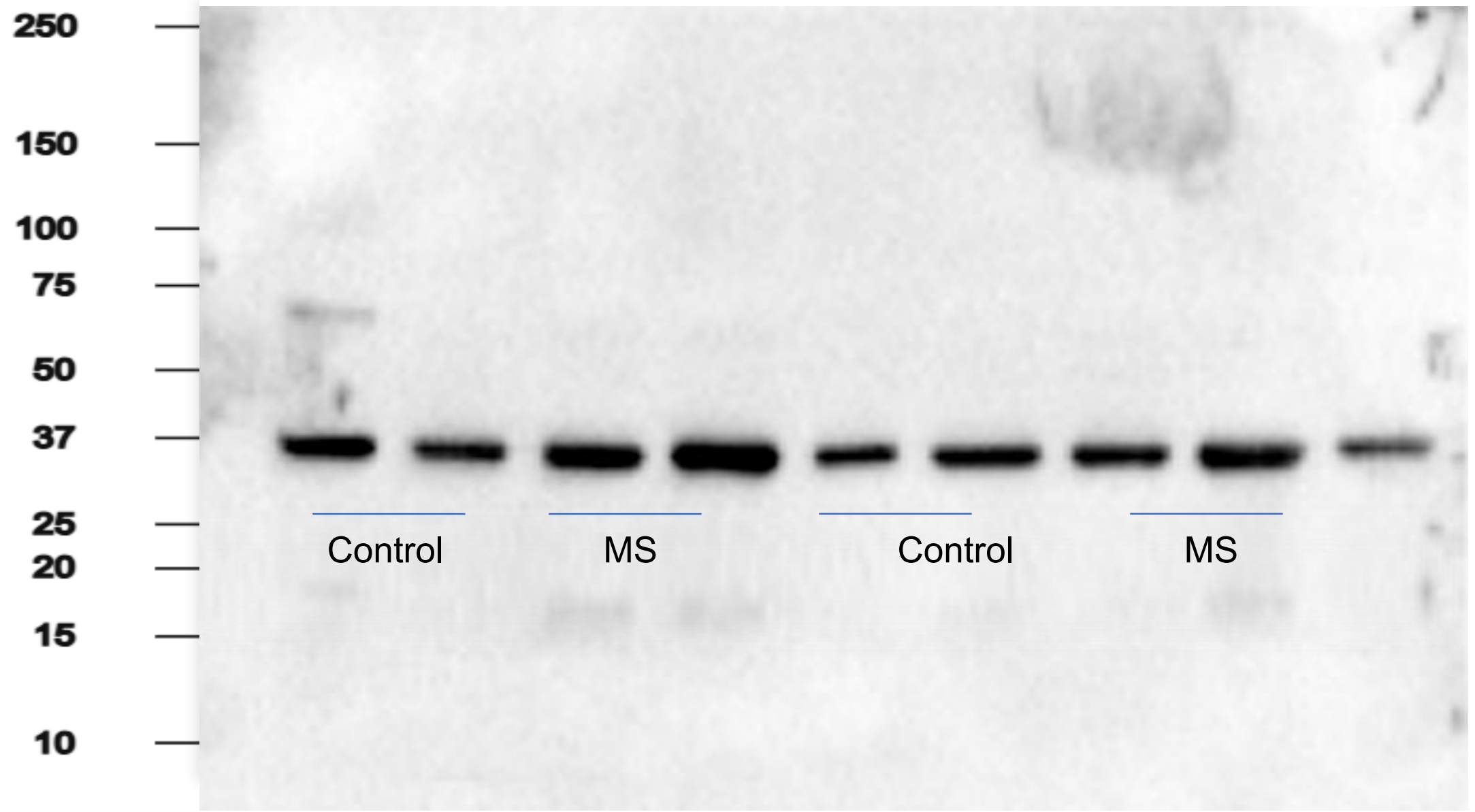
250
150
100
75
50
37
25
20
15
10
Control
MS
Control
MS

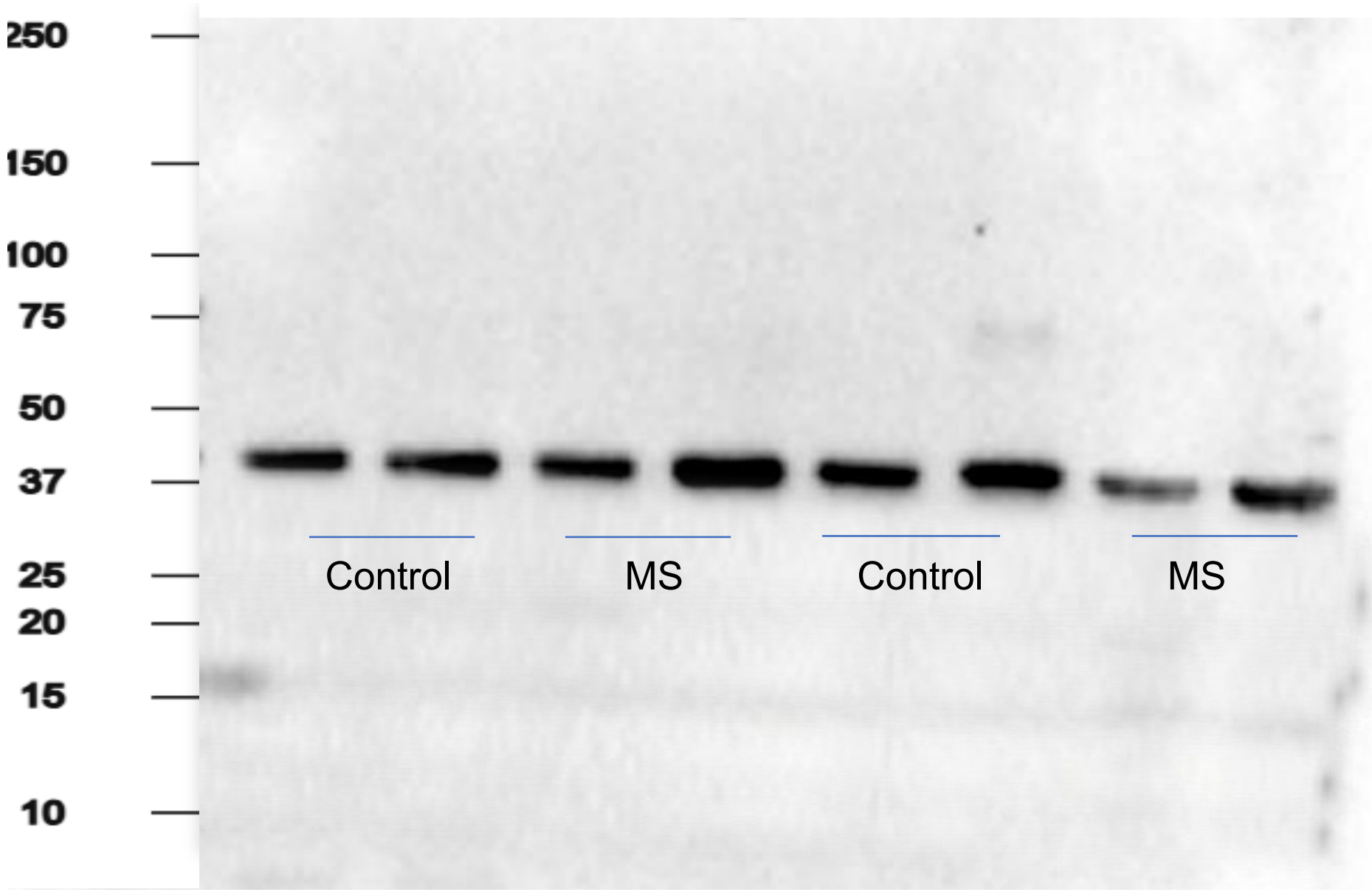
250
150
100
75
50
37
25
20
15
10
Control
MS
Control
MS

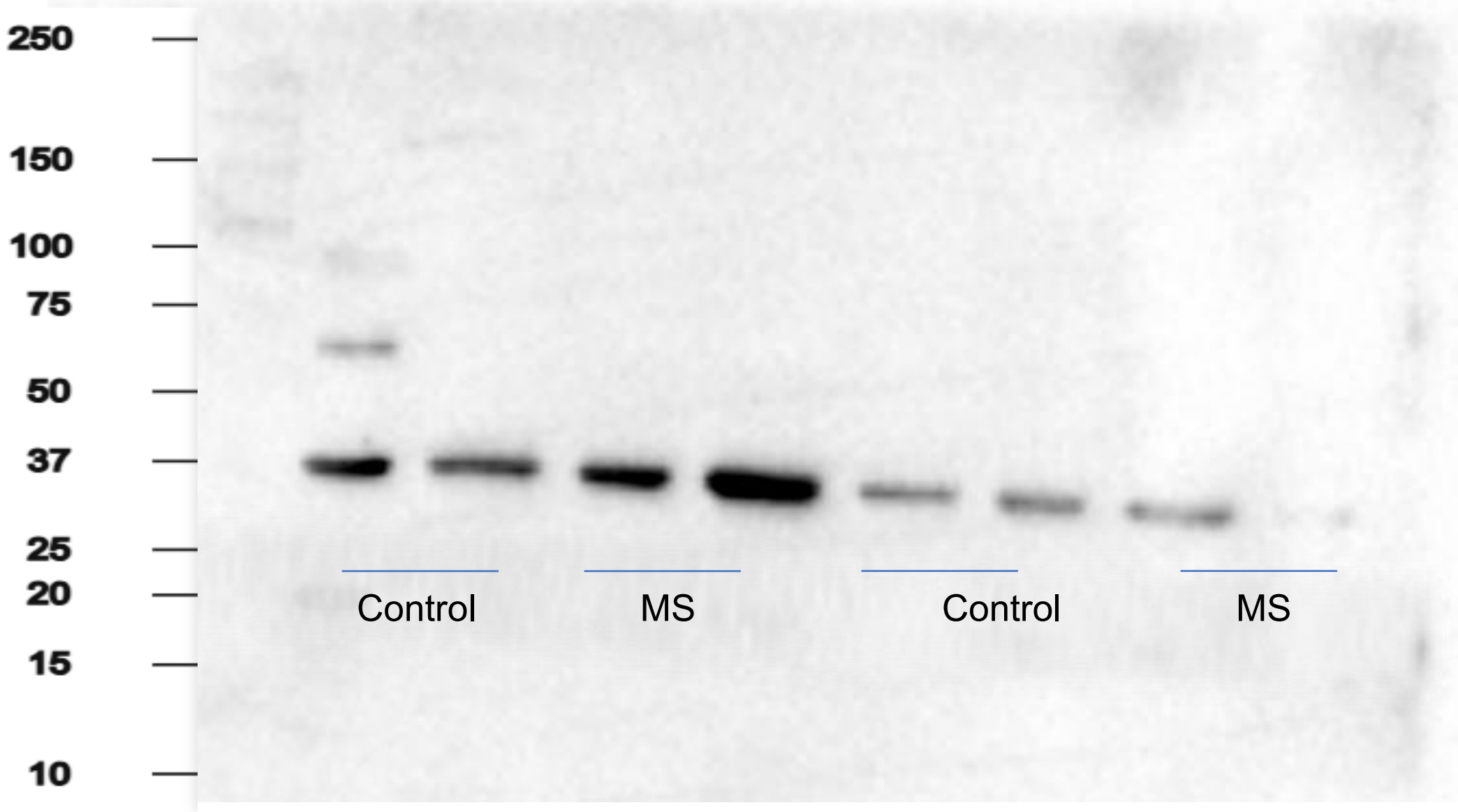
250
150
100
75
50
37
25
20
15
10
Control
MS
Control
MS

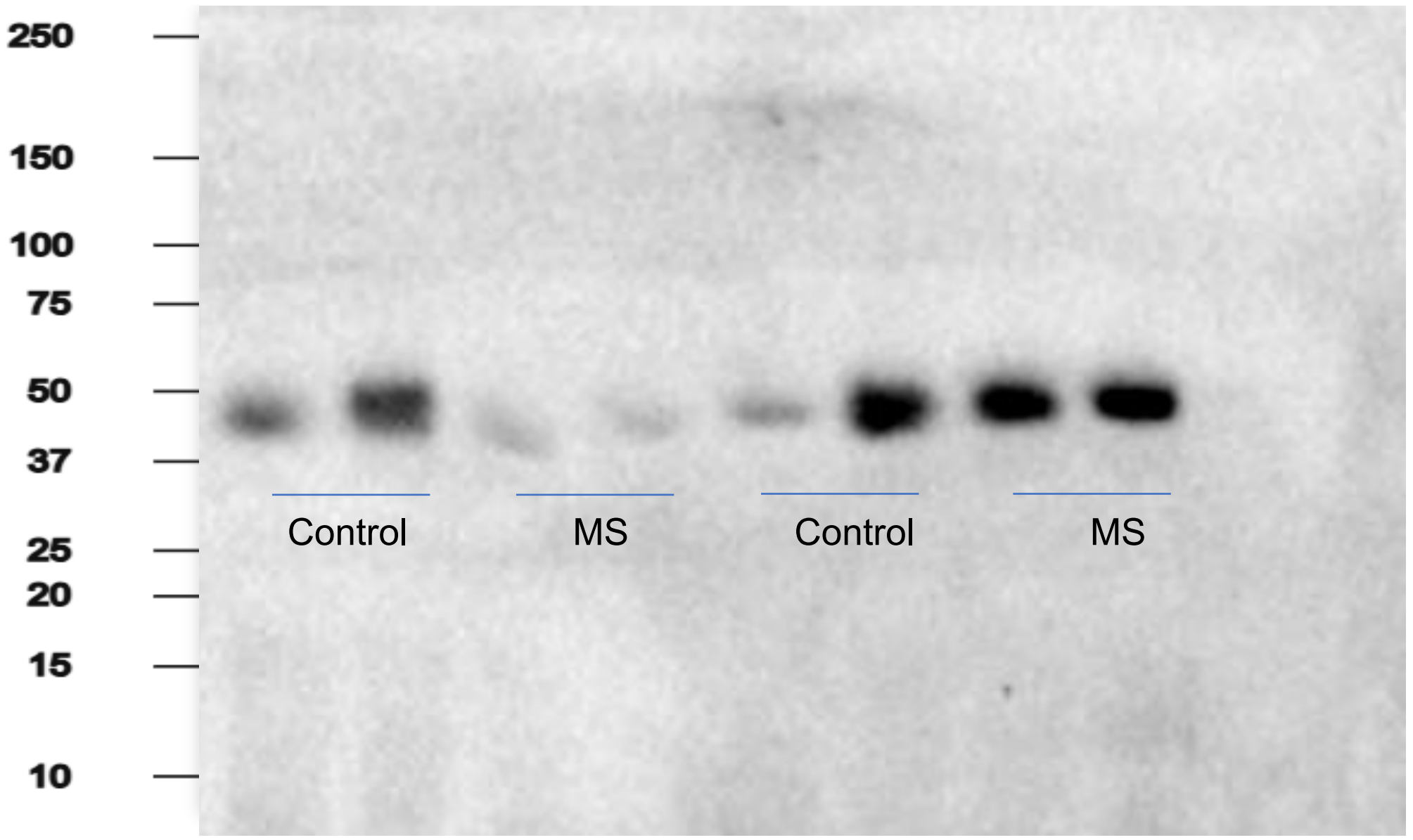
250
150
100
75
50
37
25
20
15
10
Control
MS
Control
MS

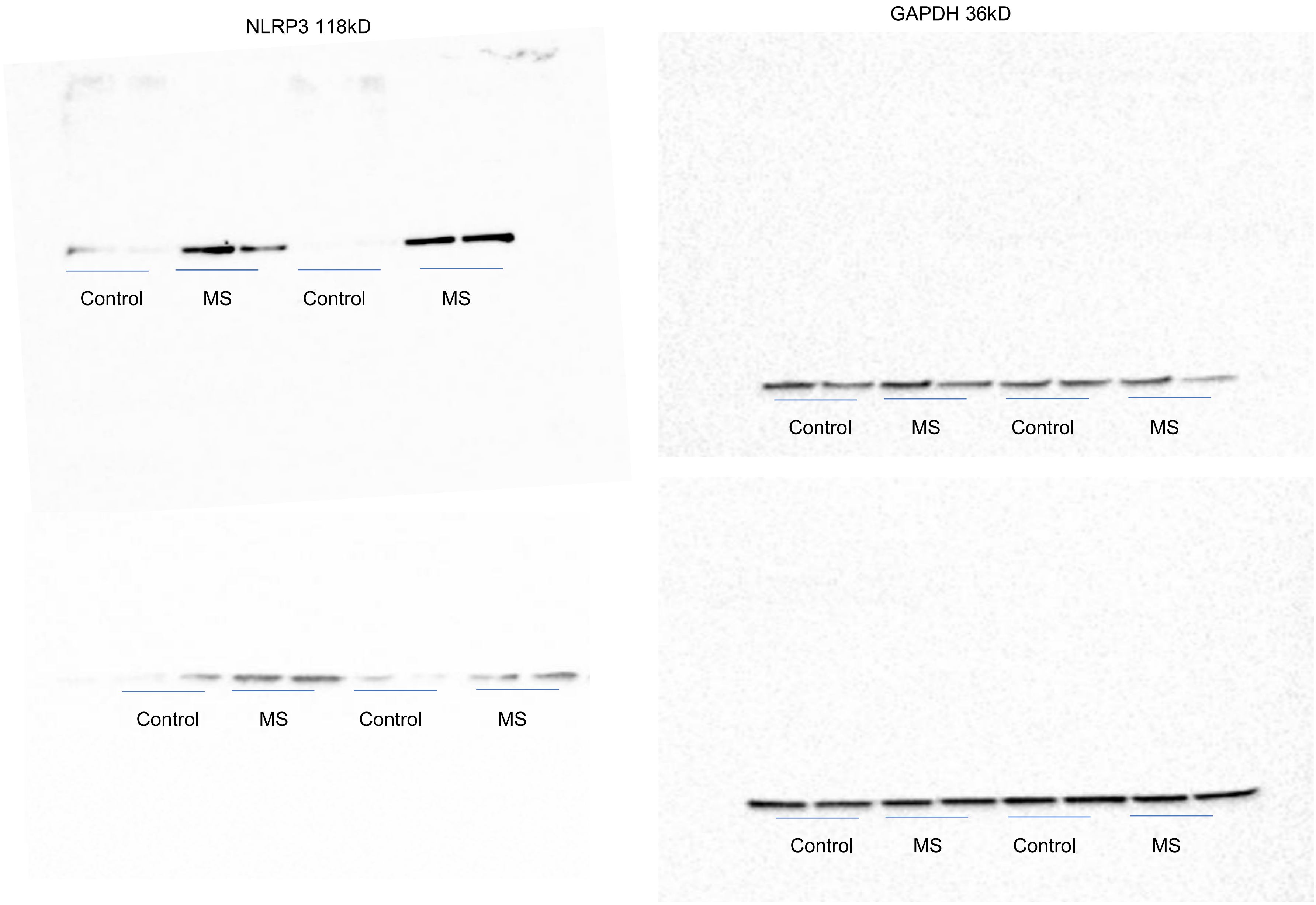
NLRP3 118kD
Control
MS
Control
MS
Control
MS
Control
MS
GAPDH 36kD
Control
MS
Control
MS
Control
MS
Control
MS
