# Supplementary material for: Associations Between Neuromuscular Fatigue, Anxiety, and Peripheral Biomarkers of Neuronal Injury, Inflammation, and Mitochondrial Dysfunction in Treatment‐Naïve Relapsing‐Remitting Multiple Sclerosis
Source: Brain Behav. 2026 Aug 25;16(8):e71707. doi: 10.1002/brb3.71707 (PMC13507521; doi:10.1002/brb3.71707)
Supplement: Supplementary file 2 [file BRB3-16-e71707-s005.pdf]

Supplementary Material – Figure 4.1 - Moderate and strong correlations among clinical and biochemical variables in untreated patients with multiple sclerosis.

Variable_1	Variable_2	Spearman_rho	Spearman_p	Direction	Strength
BDNF	NRF1	0,68872	0,0008	Positive	Strong
Catalase	TGF-B	0,60775	0,0045	Positive	Strong
S100	TGF-B	0,60451	0,0048	Positive	Strong
TNF-alpha	Sex	0,67635	0,0011	Positive	Strong
8-oxoG	TGF-B	0,47368	0,0349	Positive	Moderate
Age	IL-10	0,53440	0,0152	Positive	Moderate
Age	IL-18	0,47242	0,0354	Positive	Moderate
Age	Enolase	0,47166	0,0358	Positive	Moderate
BAX	total anxiety scale	0,50997	0,0216	Positive	Moderate
BAX	BCL-2	-0,44528	0,0491	Negative	Moderate
BCL-2	Pink	-0,55038	0,0119	Negative	Moderate
BCL-2	total anxiety scale	-0,52972	0,0163	Negative	Moderate
Enolase	BCL-2	-0,51729	0,0195	Negative	Moderate
Enolase	total anxiety scale	0,48758	0,0292	Positive	Moderate
Enolase	BAX	0,46559	0,0386	Positive	Moderate
IL-18	TFAM	-0,55334	0,0114	Negative	Moderate
NRF1	NGF	-0,46709	0,0379	Negative	Moderate
Parkin	Sex	0,46824	0,0373	Positive	Moderate
Parkin	total anxiety scale	-0,46050	0,0410	Negative	Moderate
TFAM	Parkin	0,49227	0,0275	Positive	Moderate
TNF-alpha	8-oxoG	-0,54586	0,0128	Negative	Moderate
Vitamin D	Sex	0,53761	0,0145	Positive	Moderate

This table shows the moderate and strong correlations identified in the exploratory correlation analysis. Spearman's rank correlation coefficient was used as the primary method. Correlations were classified according to the absolute Spearman's rho value as moderate when $0.40 \leq | rho | < 0.60$, strong when $0.60 \leq | rho | < 0.80$, and very strong when $| rho | \geq 0.80$. Positive rho values indicate direct associations, whereas negative rho values indicate inverse associations. Raw p-values and, when available, Benjamini–Hochberg false discovery rate-adjusted p-values are shown. Because of the exploratory nature of the analysis and the limited sample size, these findings should be interpreted as hypothesis-generating.

Supplementary Material – Figure 4.2 - P-value matrix for Spearman correlations among clinical and biochemical variables in untreated patients with multiple sclerosis.

	Age	IL-1β	IL-10	TNF-α	IL-18	NLRP3	Tbars	Carbonyl	DCF-DA	Cat	8-oxoG	SNE	BDNF	S100β	TGFβ	TFAM	NRF1	BAX	BCL-2	Parkin	Pink	NGF	Vitamin D	total anxiety scale	Sex	Fatigue
**Age**	0,000	0,227	0,015	0,857	0,035	0,832	0,304	0,194	0,155	0,864	0,859	0,036	0,761	0,852	0,718	0,400	0,776	0,159	0,578	0,222	0,355	0,483	0,788	0,091	0,418	0,448
**IL-1B**	0,227	0,000	0,130	0,286	0,295	0,539	0,556	0,705	0,326	0,418	0,561	0,110	0,246	0,677	0,283	0,434	0,787	0,107	0,454	0,443	0,427	0,311	0,552	0,930	0,097	0,201
**IL-10**	0,015	0,130	0,000	0,548	0,369	0,552	0,089	0,405	0,544	0,448	0,627	0,222	0,920	0,696	0,880	0,447	0,316	0,081	0,787	0,605	0,691	0,274	0,454	0,261	0,511	0,677
**TNF-alpha**	0,857	0,286	0,548	0,000	0,880	0,569	0,400	0,088	0,435	0,279	0,013	0,132	0,955	0,724	0,561	0,709	0,336	0,965	0,955	0,301	0,450	0,897	0,056	0,488	0,001	0,558
**IL-18**	0,035	0,295	0,369	0,880	0,000	0,710	0,932	0,691	0,346	0,114	0,995	0,346	0,376	0,985	0,960	0,011	0,840	0,340	0,840	0,380	0,870	0,261	0,636	0,535	0,465	0,100
**NLRP3**	0,832	0,539	0,552	0,569	0,710	0,000	0,645	0,618	0,600	0,285	0,115	0,830	0,920	0,214	0,083	0,495	0,474	0,126	0,811	0,860	0,446	0,556	0,578	0,334	0,716	0,450
**Tbars**	0,304	0,556	0,089	0,400	0,932	0,645	0,000	0,103	0,689	0,505	0,796	0,544	0,997	0,826	0,703	0,362	0,439	0,439	0,794	0,151	0,486	0,522	0,437	0,764	0,827	0,676
**Carbonyl**	0,194	0,705	0,405	0,088	0,691	0,618	0,103	0,000	0,104	0,454	0,050	0,148	0,128	0,600	0,796	0,762	0,965	0,556	0,316	0,222	0,574	0,433	0,146	0,374	0,511	0,145
**DCF-DA**	0,155	0,326	0,544	0,435	0,346	0,600	0,689	0,104	0,000	0,734	0,782	0,268	0,283	0,135	0,710	0,289	0,955	0,782	0,885	0,246	0,280	0,185	0,105	0,269	0,114	0,934
**Catalase**	0,864	0,418	0,448	0,279	0,114	0,285	0,505	0,454	0,734	0,000	0,237	0,620	0,947	0,180	0,004	0,534	0,962	0,114	0,166	0,895	0,389	0,460	0,350	0,356	0,180	0,676
**8-oxoG**	0,859	0,561	0,627	0,013	0,995	0,115	0,796	0,050	0,782	0,237	0,000	0,691	0,970	0,116	0,035	0,621	0,274	0,195	0,454	0,645	0,801	0,689	0,591	0,360	0,379	0,803
**Enolase**	0,036	0,110	0,222	0,132	0,346	0,830	0,544	0,148	0,268	0,620	0,691	0,000	0,086	0,450	0,821	0,203	0,148	0,039	0,020	0,356	0,821	0,293	0,450	0,029	0,068	0,271
**BDNF**	0,761	0,246	0,920	0,955	0,376	0,920	0,997	0,128	0,283	0,947	0,970	0,086	0,000	0,506	0,622	0,579	0,001	0,062	0,865	0,945	0,072	0,518	0,431	0,345	0,134	0,677
**S100**	0,852	0,677	0,696	0,724	0,985	0,214	0,826	0,600	0,135	0,180	0,116	0,450	0,506	0,000	0,005	0,599	0,552	0,947	0,207	0,596	0,398	0,291	0,329	0,121	0,511	0,677
**TGF-B**	0,718	0,283	0,880	0,561	0,960	0,083	0,703	0,796	0,710	0,004	0,035	0,821	0,622	0,005	0,000	0,882	0,719	0,142	0,705	0,915	0,875	0,813	0,539	0,782	0,236	0,271
**TFAM**	0,400	0,434	0,447	0,709	0,011	0,495	0,362	0,762	0,289	0,534	0,621	0,203	0,579	0,599	0,882	0,000	0,082	0,521	0,493	0,027	0,323	0,081	0,127	0,597	0,398	0,200
**NRF1**	0,776	0,787	0,316	0,336	0,840	0,474	0,439	0,965	0,955	0,962	0,274	0,148	0,001	0,552	0,719	0,082	0,000	0,221	0,920	0,474	0,609	0,038	0,801	0,661	0,465	0,450
**BAX**	0,159	0,107	0,081	0,965	0,340	0,126	0,439	0,556	0,782	0,114	0,195	0,039	0,062	0,947	0,142	0,521	0,221	0,000	0,049	0,835	0,537	0,438	0,818	0,022	0,464	0,100
**BCL-2**	0,578	0,454	0,787	0,955	0,840	0,811	0,794	0,316	0,885	0,166	0,454	0,020	0,865	0,207	0,705	0,493	0,920	0,049	0,000	0,654	0,012	0,380	0,578	0,016	1,000	0,100
**Parkin**	0,222	0,443	0,605	0,301	0,380	0,860	0,151	0,222	0,246	0,895	0,645	0,356	0,945	0,596	0,915	0,027	0,474	0,835	0,654	0,000	0,169	0,782	0,394	0,041	0,037	0,100
**Pink**	0,355	0,427	0,691	0,450	0,870	0,446	0,486	0,574	0,280	0,389	0,801	0,821	0,072	0,398	0,875	0,323	0,609	0,537	0,012	0,169	0,000	0,794	0,591	0,293	0,716	0,271
**NGF**	0,483	0,311	0,274	0,897	0,261	0,556	0,522	0,433	0,185	0,460	0,689	0,293	0,518	0,291	0,813	0,081	0,038	0,438	0,380	0,782	0,794	0,000	0,794	0,427	0,662	0,100
**Vitamin D**	0,788	0,552	0,454	0,056	0,636	0,578	0,437	0,146	0,105	0,350	0,591	0,450	0,431	0,329	0,539	0,127	0,801	0,818	0,578	0,394	0,591	0,794	0,000	0,977	0,014	0,100
**total anxiety scale**	0,091	0,930	0,261	0,488	0,535	0,334	0,764	0,374	0,269	0,356	0,360	0,029	0,345	0,121	0,782	0,597	0,661	0,022	0,016	0,041	0,293	0,427	0,977	0,000	0,105	0,100
**Sex**	0,418	0,097	0,511	0,001	0,465	0,716	0,827	0,511	0,114	0,180	0,379	0,068	0,134	0,511	0,236	0,398	0,465	0,464	1,000	0,037	0,716	0,662	0,014	0,105	0,000	0,331
**Fatigue**	0,448	0,201	0,677	0,558	0,100	0,450	0,676	0,145	0,934	0,676	0,803	0,271	0,677	0,677	0,271	0,200	0,450	0,100	0,100	0,100	0,271	0,100	0,100	0,100	0,331	0,000

This table presents the corresponding p-values for the Spearman's rank correlation coefficients shown in figure 4.2. Each p-value refers to the statistical significance of the pairwise association between the respective variables. Because multiple pairwise correlations were tested, the results should be interpreted cautiously. The correlation analysis was exploratory and intended to identify potential hypothesis-generating associations among clinical and biochemical variables.

Supplementary Material – Figure 4.3 - Complete Spearman's rho correlation matrix among clinical and biochemical variables in untreated patients with multiple sclerosis.

	Age	IL-1β	IL-10	TNF-α	IL-18	NLRP3	Tbars	Carbonyl	DCF-DA	Cat	8-oxoG	SNE	BDNF	S100β	TGFβ	TFAM	NRF1	BAX	BCL-2	Parkin	Pink	NGF	Vitamin D	total anxiety scale	Sex	Fatigue
**Age**	1,000	-0,283	0,534	-0,043	0,472	-0,051	0,242	-0,303	0,330	0,041	-0,042	0,472	-0,073	0,045	0,086	-0,199	0,068	0,327	-0,132	-0,286	0,218	-0,166	0,064	0,388	-0,192	0,180
**IL-1B**	-0,283	1,000	-0,350	0,251	-0,247	0,146	0,140	0,090	0,232	0,192	0,138	-0,368	0,272	0,099	0,253	0,185	0,065	-0,371	0,177	0,182	-0,188	0,238	0,141	-0,021	0,382	-0,298
**IL-10**	0,534	-0,350	1,000	-0,143	0,212	-0,141	-0,390	0,197	0,144	-0,180	-0,116	0,286	-0,024	0,093	-0,036	-0,180	0,236	0,399	-0,065	-0,123	0,095	-0,257	0,177	0,264	-0,156	-0,099
**TNF-alpha**	-0,043	0,251	-0,143	1,000	-0,036	-0,135	0,199	-0,391	-0,185	-0,254	-0,546	-0,349	0,014	-0,084	-0,138	0,089	0,227	0,011	-0,014	0,244	-0,179	0,031	0,435	-0,165	0,676	-0,139
**IL-18**	0,472	-0,247	0,212	-0,036	1,000	-0,089	0,020	-0,095	0,223	-0,365	-0,002	0,223	-0,209	-0,005	-0,012	-0,553	0,048	0,225	-0,048	-0,208	-0,039	-0,264	-0,113	0,147	-0,173	0,378
**NLRP3**	-0,051	0,146	-0,141	-0,135	-0,089	1,000	-0,110	-0,119	0,125	0,251	0,364	0,051	0,024	0,290	0,397	0,162	0,170	-0,354	-0,057	0,042	-0,180	0,140	-0,132	0,228	-0,087	0,179
**Tbars**	0,242	0,140	-0,390	0,199	0,020	-0,110	1,000	-0,375	0,096	0,158	0,062	-0,144	0,001	0,053	0,091	-0,215	-0,184	-0,183	0,062	-0,333	0,165	0,152	0,184	0,072	0,052	0,100
**Carbonyl**	-0,303	0,090	0,197	-0,391	-0,095	-0,119	-0,375	1,000	-0,374	-0,178	0,444	-0,335	0,352	0,125	-0,062	0,072	0,011	-0,140	0,236	0,286	0,134	-0,186	0,337	-0,210	0,156	-0,338
**DCF-DA**	0,330	0,232	0,144	-0,185	0,223	0,125	0,096	-0,374	1,000	0,081	-0,066	0,260	-0,253	-0,346	0,089	-0,250	-0,014	-0,066	-0,035	-0,272	-0,254	0,309	-0,373	0,260	-0,364	-0,020
**Catalase**	0,041	0,192	-0,180	-0,254	-0,365	0,251	0,158	-0,178	0,081	1,000	0,277	0,118	0,016	0,312	0,608	0,148	-0,011	-0,365	0,322	-0,032	-0,204	-0,175	-0,220	-0,218	-0,312	-0,100
**8-oxoG**	-0,042	0,138	-0,116	-0,546	-0,002	0,364	0,062	0,444	-0,066	0,277	1,000	0,095	-0,009	0,362	0,474	0,118	-0,257	-0,302	0,177	-0,110	0,060	0,096	0,128	0,216	-0,208	-0,060
**Enolase**	0,472	-0,368	0,286	-0,349	0,223	0,051	-0,144	-0,335	0,260	0,118	0,095	1,000	-0,394	0,179	0,054	0,297	-0,335	0,466	-0,517	-0,218	0,054	0,247	-0,179	0,488	-0,416	0,259
**BDNF**	-0,073	0,272	-0,024	0,014	-0,209	0,024	0,001	0,352	-0,253	0,016	-0,009	-0,394	1,000	0,158	-0,117	-0,132	0,689	-0,425	0,041	-0,017	0,411	-0,153	0,186	-0,223	0,347	-0,099
**S100**	0,045	0,099	0,093	-0,084	-0,005	0,290	0,053	0,125	-0,346	0,312	0,362	0,179	0,158	1,000	0,605	0,125	0,141	0,016	-0,295	-0,126	0,200	-0,248	0,230	0,358	-0,156	0,099
**TGF-B**	0,086	0,253	-0,036	-0,138	-0,012	0,397	0,091	-0,062	0,089	0,608	0,474	0,054	-0,117	0,605	1,000	-0,035	-0,086	-0,340	0,090	-0,026	-0,038	-0,056	-0,146	0,066	-0,277	-0,259
**TFAM**	-0,199	0,185	-0,180	0,089	-0,553	0,162	-0,215	0,072	-0,250	0,148	0,118	0,297	-0,132	0,125	-0,035	1,000	-0,399	0,152	-0,163	0,492	-0,233	0,400	0,353	0,126	0,200	-0,299
**NRF1**	0,068	0,065	0,236	0,227	0,048	0,170	-0,184	0,011	-0,014	-0,011	-0,257	-0,335	0,689	0,141	-0,086	-0,399	1,000	-0,287	0,024	-0,170	0,122	-0,467	0,060	-0,105	0,173	0,179
**BAX**	0,327	-0,371	0,399	0,011	0,225	-0,354	-0,183	-0,140	-0,066	-0,365	-0,302	0,466	-0,425	0,016	-0,340	0,152	-0,287	1,000	-0,445	0,050	0,147	-0,184	-0,055	0,510	-0,173	0,378
**BCL-2**	-0,132	0,177	-0,065	-0,014	-0,048	-0,057	0,062	0,236	-0,035	0,322	0,177	-0,517	0,041	-0,295	0,090	-0,163	0,024	-0,445	1,000	0,107	-0,550	-0,208	0,132	-0,530	0,000	-0,378
**Parkin**	-0,286	0,182	-0,123	0,244	-0,208	0,042	-0,333	0,286	-0,272	-0,032	-0,110	-0,218	-0,017	-0,126	-0,026	0,492	-0,170	0,050	0,107	1,000	-0,320	-0,066	0,202	-0,460	0,468	-0,378
**Pink**	0,218	-0,188	0,095	-0,179	-0,039	-0,180	0,165	0,134	-0,254	-0,204	0,060	0,054	0,411	0,200	-0,038	-0,233	0,122	0,147	-0,550	-0,320	1,000	-0,062	-0,128	0,248	0,087	0,259
**NGF**	-0,166	0,238	-0,257	0,031	-0,264	0,140	0,152	-0,186	0,309	-0,175	0,096	0,247	-0,153	-0,248	-0,056	0,400	-0,467	-0,184	-0,208	-0,066	-0,062	1,000	0,062	0,188	0,104	-0,378
**Vitamin D**	0,064	0,141	0,177	0,435	-0,113	-0,132	0,184	0,337	-0,373	-0,220	0,128	-0,179	0,186	0,230	-0,146	0,353	0,060	-0,055	0,132	0,202	-0,128	0,062	1,000	0,007	0,538	-0,378
**total anxiety scale**	0,388	-0,021	0,264	-0,165	0,147	0,228	0,072	-0,210	0,260	-0,218	0,216	0,488	-0,223	0,358	0,066	0,126	-0,105	0,510	-0,530	-0,460	0,248	0,188	0,007	1,000	-0,373	0,378
**Sex**	-0,192	0,382	-0,156	0,676	-0,173	-0,087	0,052	0,156	-0,364	-0,312	-0,208	-0,416	0,347	-0,156	-0,277	0,200	0,173	-0,173	0,000	0,468	0,087	0,104	0,538	-0,373	1,000	-0,229
**Fatigue**	0,180	-0,298	-0,099	-0,139	0,378	0,179	0,100	-0,338	-0,020	-0,100	-0,060	0,259	-0,099	0,099	-0,259	-0,299	0,179	0,378	-0,378	-0,378	0,259	-0,378	-0,378	0,378	-0,229	1,000

This table presents the complete matrix of Spearman's rank correlation coefficients for all pairwise associations among clinical and biochemical variables. Positive values indicate positive correlations, whereas negative values indicate inverse correlations. Values closer to +1 or -1 indicate stronger correlations, while values close to 0 indicate weak or absent monotonic associations. Spearman's correlation was selected as the primary method because of the small sample size and the non-normal distribution of several variables.
