# Supplementary material for: Associations Between Neuromuscular Fatigue, Anxiety, and Peripheral Biomarkers of Neuronal Injury, Inflammation, and Mitochondrial Dysfunction in Treatment‐Naïve Relapsing‐Remitting Multiple Sclerosis
Source: Brain Behav. 2026 Aug 25;16(8):e71707. doi: 10.1002/brb3.71707 (PMC13507521; doi:10.1002/brb3.71707)
Supplement: Supplementary file 3 [file BRB3-16-e71707-s004.docx]

| **Descriptor** | **Control** | **SM** |
| --- | --- | --- |
| **Sex** |  |  |
| M | 10 (50%) | 10 (50%) |
| F | 10 (50%) | 10 (50%) |
| **Age** |  |  |
| 20 - 30 | 4 (20%) | 4 (20%) |
| 31 – 40 | 9 (45%) | 9 (45%) |
| 41 - 50 | 7 (35%) | 7 (35%) |
| **ETHNICITY** |  |  |
| White | 15 (75%) | 12 (60%) |
| Brown | 5 (25%) | 8 (40%) |
| **LEVEL OF EDUCATION** |  |  |
| Complete primary education | - | - |
| Incomplete primary education | 4 (20%) | 4 (20%) |
| Complete high school | 8 (40%) | 5 (25%) |
| Incomplete high school | 1 (5%) | 2 (10%) |
| Complete higher education | 6 (30%) | 6 (30%) |
| Incomplete higher education | 1 (5%) | 3 (15%) |
| **MEDICAL SUPERVISION** |  |  |
| Yes | 3 (15%) | 16 (80%) |
| No | 17 (85%) | 4 (20%) |
| **PSYCHOTHERAPIST SUPERVISION** |  |  |
| Yes | 3 (15%) | 11 (55%) |
| No | 17 (85%) | 9 (45%) |
| **REPORTS CHANGES IN MENTAL HEALTH DUE TO THE DISEASE** |  |  |
| Yes | - | 20 (100%) |
| No | - | - |
| **SYMTOMS** |  |  |
| Low sight  Weakness in limbs  Numbness or tingling in the body  Double Vision  Difficulty speaking or swallowing  Fatigue  Urinary incontinence  Imbalance | -  -  -  -  -  -  -  -  - | 4 (20%)  3 (15%)  2 (10%)  1 (5%)  10 (50%)  18 (90%)  4 (20%)  8 (40%) |
|  |  |  |
